# Use of a human immortalized microglia cell line to study recognition, phagocytosis, and intracellular survival of *Cryptococcus neoformans*

**DOI:** 10.1128/msphere.00838-25

**Published:** 2026-01-30

**Authors:** Robbi L. Ross, Kassandra Arias-Parbul, Zane M. Douglass, Katrina L. Adams, Felipe H. Santiago-Tirado

**Affiliations:** 1 Department of Biological Sciences, University of Notre Dame 6111, Notre Dame, Indiana, USA; 2 Integrated Biomedical Sciences, University of Notre Dame 6111, Notre Dame, Indiana, USA; 3 Center for Stem Cells and Regenerative Medicine, University of Notre Dame 6111, Notre Dame, Indiana, USA; 4 Eck Institute for Global Health, University of Notre Dame 6111, Notre Dame, Indiana, USA; 5 Warren Center for Drug Discovery, University of Notre Dame 6111, Notre Dame, Indiana, USA; University of Michigan Michigan Medicine, Ann Arbor, Michigan, USA

**Keywords:** *Cryptococcus neoformans*, microglia, cryptococcal meningitis, C20 cells

## Abstract

*Cryptococcus neoformans,* the etiological agent of cryptococcal meningitis (CM) is a globally distributed environmental yeast that mainly causes infections in immunocompromised individuals. Particularly in low-resource countries, the mortality rate of CM can reach 81% and accounts for ~19% of HIV/AIDS-related deaths each year. In immunocompromised individuals, once inhaled, *C. neoformans* escapes from the lungs and disseminates with special predilection for the central nervous system (CNS). Once in the brain, *C. neoformans* interacts with microglia, the tissue-resident macrophages of the CNS. Previous studies indirectly showed that microglia are ineffective at controlling this fungal infection. The mechanisms underlying this fungal survival and proliferation within the CNS, however, remain unclear. In this study, we use and validate the C20 immortalized human microglia cell line to study cryptococcal-microglia interactions. We show that microglia have limited phagocytic activity that is specific to *C. neoformans* and partly dependent on cryptococcal antiphagocytic proteins that alter cell size and cell wall structure. We also show that human microglia respond to cryptococcal strains differently than peripheral macrophages. Further, we show that human microglia are ineffective at killing phagocytosed *C. neoformans*, and that this could be due to the ability of this yeast to disrupt phagosome maturation and induce phagosome membrane damage in these cells. These findings provide us with fundamental knowledge regarding cryptococcal pathogenesis in the CNS, specifically the insight into how *C. neoformans* is recognized by microglia under different conditions and demonstrate the usefulness of C20 cells to further study how this yeast survives and replicates within the CNS environment.

**IMPORTANCE:**

While *Cryptococcus neoformans* is acquired through inhalation, the fatal pathology of cryptococcal infection occurs when the yeast disseminates to the central nervous system (CNS) and causes cryptococcal meningitis. Microglia are the first immune cells that *C. neoformans* will encounter once it reaches the CNS, and they are the largest population of macrophages in the brain. While microglia are professional phagocytes, they are unable to control *C. neoformans* infection. The mechanisms behind uncontrolled growth of *C. neoformans* within the CNS remain understudied, partly due to incomplete knowledge regarding microglia-cryptococcal interactions. This study provides fundamental knowledge into these interactions and establishes a powerful model to specifically study how *C. neoformans* is recognized by microglia and how cryptococcal phagosomes mature in these phagocytes. This work opens new avenues of research to further our understanding of cryptococcal-host interactions, which can be leveraged to develop more effective therapeutics for cryptococcal meningitis.

## INTRODUCTION

Of the four to five million fungal species worldwide (1), few are frequent pathogens of humans (2). However, this small number of fungi causes over 80 million invasive infections annually, resulting in 3.8 million deaths (3). One of the most common and challenging of these infections is caused by the environmental yeast *Cryptococcus neoformans* (4). *C. neoformans* is globally distributed; hence, we are all frequently exposed, and it is responsible for about 147,000 annual deaths, mostly in immunocompromised individuals (3, 5). Desiccated yeasts and spores are inhaled and become lodged in the lungs. In immunocompetent individuals, the lung-resident macrophages (alveolar macrophages, AMs) are able to control or clear the infection. However, under immunocompromised conditions, the yeast disseminates from the lungs with special predilection for the central nervous system (CNS), resulting in lethal meningitis or meningoencephalitis (commonly known simply as cryptococcal meningitis [CME]). CME is a leading cause of death in patients with HIV/AIDS and has a general mortality rate of 76% (3, 5). The interactions of immune cells, particularly tissue-resident macrophages, with this fungus are critical determinants of disease outcome (6). Tissue-resident macrophages are in every organ and function in maintaining homeostasis and immune surveillance, providing rapid defense against invading pathogens. Within the CNS, there are several tissue-resident macrophages present in different anatomical locations. The most numerous and well-studied of these include microglia, which are in the brain parenchyma (7).

As the brain’s resident immune cells, microglia have several functions, including synapse formation and degradation, engulfment of apoptotic neurons and neurotransmitters, and immune surveillance (8). When pathogens, including bacteria, viruses, fungi, and parasites, invade the CNS, or when there is mechanical trauma, microglia exhibit rapid responses to clear the invading pathogens or tissue damage, respectively. In most cases of CNS infection, microglia are the first immune cells encountered by pathogens and have been shown to be critical in controlling several of them, highlighting the importance of the microglia-pathogen interactions (9, 10).

While the lethal pathology of cryptococcal infection occurs in the brain, the interactions of *C. neoformans* with microglia remain understudied. However, it has been shown that microglia play a permissive, rather than protective, role during cryptococcal infection. *In vivo* experiments have shown that rapid expansion of *C. neoformans* in the brain does not immediately activate murine microglia nor cause them to upregulate MHC class II expression (11, 12). Additionally, *ex vivo* and *in vivo* histological analyses show that *C. neoformans* survives and replicates within human microglia, implying limited microglia fungicidal activity (13–15). The mechanisms responsible for this uncontrolled growth of *C. neoformans* and lack of microglial activation remain unclear, mostly because of a lack of physiologically relevant *in vitro* models that would allow for mechanistic studies.

In most instances, after recognition and phagocytosis by microglia, a pathogen will reside in a phagosome that undergoes a series of maturation steps, becoming a microbicidal phagolysosome and contributing to control of the infection. However, indirect evidence implies that *C. neoformans* resides in the microglial phagolysosome and resumes intracellular replication (16). In peripheral macrophages, *C. neoformans* manipulates phagosome maturation by altering phagosome acidification, inducing membrane damage, and impairing fungicidal activities (17–24). Despite this, studies extending these findings to microglia are lacking. Furthermore, the similarities and differences regarding cryptococcal interactions between microglia and peripheral macrophages remain understudied. Overall, the factors that influence recognition and engulfment of *C. neoformans* by microglia, and how these subsequently alter phagosome maturation and fungicidal activity, are not well studied.

We set out to study the interactions between *C. neoformans* and microglia before and after engulfment and define how these interactions compare with those observed in peripheral macrophages. We predominantly studied these interactions using the immortalized human microglia cell line C20, which has recently been developed and used to study a variety of microglia-HIV interactions, as well as microglia cellular processes during neuroinflammation (25–29). This cell line, which is adult-derived and immortalized via SV40 and hTERT transfection, shows high expression of microglia-specific surface markers, and its RNA profile, morphology, and immune-related function highlight their human origin and purity, as it has been compared to both primary human and rodent-derived microglia (26). These studies highlight the translatability of C20 cells to primary human microglia, and our findings show that the C20 cell line is comparable to primary human microglia and is a useful tool for studying cryptococcal-microglia interactions. Furthermore, we show that *C. neoformans* evades phagocytosis by microglia, partly due to antiphagocytic cryptococcal proteins, in a manner independent of that shown in other macrophage-cryptococcal studies. Additionally, we show that the ability of *C. neoformans* to be engulfed at higher rates by microglia could be related to cell size and cell wall modifications, subsequently altering microglial phagosome maturation. We also show that the ability of *C. neoformans* to survive within human microglia could be due to delayed phagosome maturation and fungal-induced phagosomal membrane damage. Overall, we demonstrate the utility of C20 cells to study the cryptococcal-microglia interactions, allowing for many new lines of investigation into this important topic.

## RESULTS

### Microglia have limited phagocytic and fungicidal activity that is specific to *C. neoformans*

**Fig 1:**
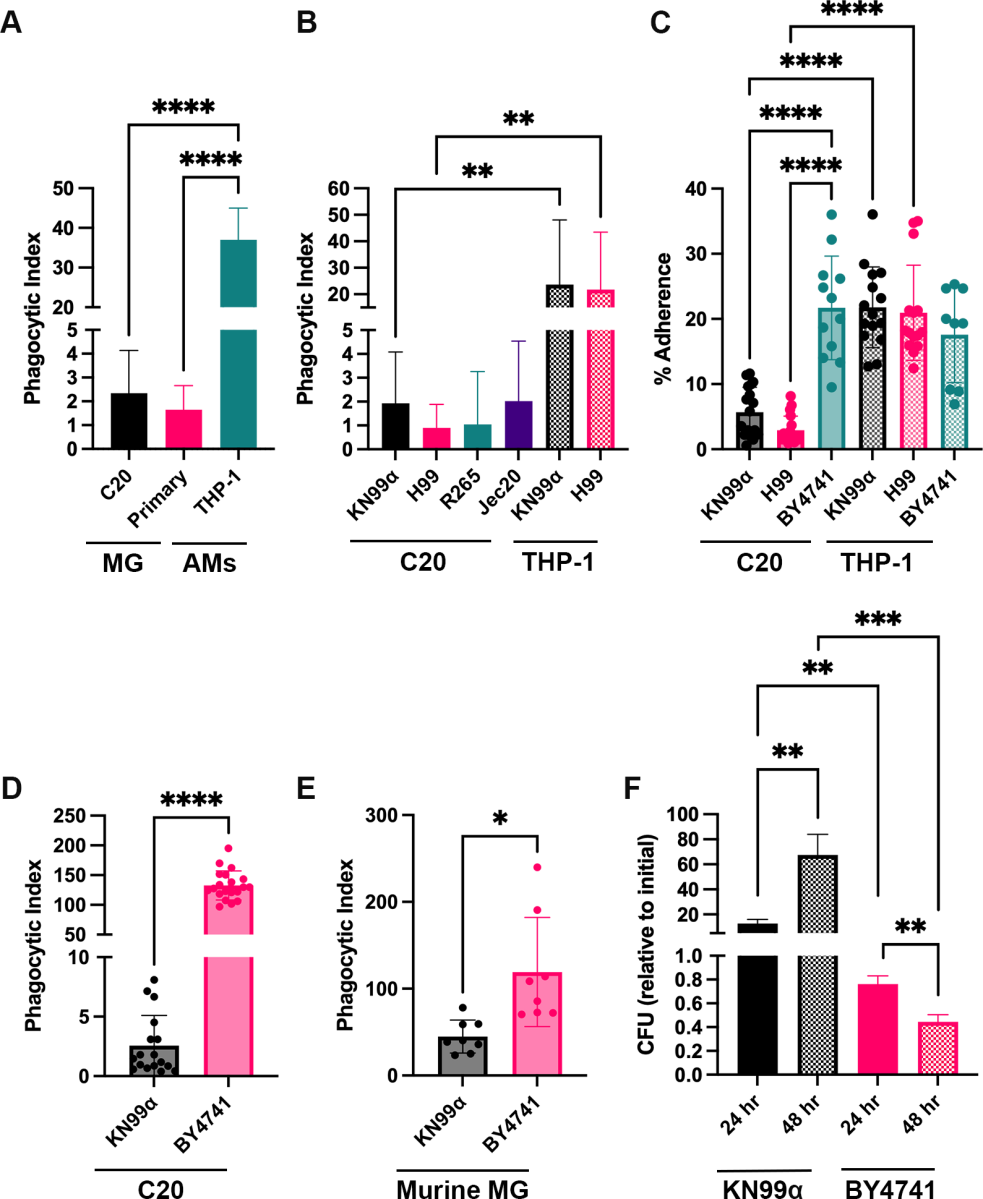
Human microglia are defective at phagocytosing *C. neoformans* and permissive to its intracellular replication. (**A**) Two types of human microglia (MG) were tested against THP-1 cells, a model of alveolar macrophages (AMs). mCherry-expressing KN99α was serum-opsonized and incubated with the different macrophages at an MOI of 20:1 for 3 h. Imaging was performed using automated microscopy and was analyzed using a CellProfiler pipeline. Phagocytic index represents the number of internalized fungi per 100 macrophages. Values represent the mean ± SD from three biological replicates. Significance was determined using one-way ANOVA with multiple comparisons (Brown-Forsythe and Welch’s corrected), ****, *P* < 0.0001. (**B**) Uptake assay testing engulfment of different cryptococcal strains (KN99α, H99, R265, Jec20) by C20 or THP-1 cells. KN99α and H99 are *C. neoformans* serotype A, Jec20 is *C. neoformans* serotype D, and R265 is *C. gattii* (all recently reclassified as different species) (30). Fungal strains were stained with lucifer yellow, serum-opsonized, and incubated with different macrophages at an MOI of 20:1 for 3 h. Imaging and analysis were performed as above. Values represent the mean ± SD from three biological replicates. Significance was determined using one-way ANOVA with multiple comparisons (Brown-Forsythe and Welch’s corrected), **, *P* < 0.01. (**C**) Assay measuring the percent adherence of different fungal strains (KN99α, H99, BY4741) to C20 and THP-1 cells. Fungal strains were incubated with the macrophages for 45 min at an MOI of 1:1. Fungi were plated before addition to the plates and after 45 min of coincubation, and colony-forming units (CFUs) were compared to obtain percent-adherence. Values represent the mean ± SD from four biological replicates. Significance was determined using one-way ANOVA with multiple comparisons (Brown-Forsythe and Welch’s corrected), ****, *P* < 0.0001. Uptake assays using C20 immortalized human microglia (**D**) and primary murine microglia (**E**) to test engulfment of KN99α and BY4741. mCherry-expressing fungi were serum-opsonized and incubated with the different microglia for 3 h at an MOI of 20:1. Imaging and analysis were performed as above. Values represent the mean ± SD of three (**D**) or two (**E**) biological replicates. Significance was determined using Welch’s t test, *, *P* < 0.05; ****, *P* < 0.0001. (**F**) *In vitro* survival of KN99α and BY4741 in C20 cells. Fungi and C20 cells were coincubated for 3 h, at which point one-third of the samples were lysed to determine intracellular fungi, and the other two-thirds were grown for 24 h and 48 h, after which they were also lysed. Shown are the CFUs that were obtained at each time point, normalized to the CFUs of initial engulfment (3 h). Values represent the mean ± SEM from 10 biological replicates. Significance was determined using one-way ANOVA with multiple comparisons (Brown-Forsythe and Welch’s corrected), **, *P* < 0.005; ***, *P* < 0.0005.

To begin studying cryptococcal-microglia interactions, we looked at the ability of C20 and PMA-differentiated THP-1 cells, models for primary human microglia (MG) and AMs, respectively, to engulf the wild-type (WT) *C. neoformans* strain KN99α (Fig. 1A). The phagocytic index (PI), which represents the number of internalized fungi per 100 macrophages, is comparable between C20 cells and primary human MG, at 2.3 and 1.7, respectively. Notably, both types of human MG have a significantly lower PI compared to that of AMs, which is 37. We wanted to confirm that this result was not dependent on the KN99α strain, so we tested the ability of C20 and THP-1 cells to engulf various strains of the *Cryptococcus* species complex (H99, R265, and Jec20) (30). As shown, C20 cells have poor engulfment of all cryptococcal strains tested relative to THP-1 cells (Fig. 1B). To determine if this lack of phagocytic activity seen with the human MG is an inherent difference between MG and AMs, we assessed the ability of C20 cells to engulf BY4741, a common *Saccharomyces cerevisiae* laboratory strain, which is non-pathogenic but similar in size and morphology to *C. neoformans* (Fig. 1D). Interestingly, C20 cells have a significantly higher PI for BY4741 (average PI = 132.6) than for KN99α (average PI = 2.56). This indicates that the lack of phagocytic activity from the human MG for *C. neoformans* (Fig. 1A and B) is fungal-dependent and not MG specific. We also saw the same result with primary murine MG, where they engulf BY4741 to a significantly higher degree than KN99α (average PIs of 119.2 and 44.9, respectively; Fig. 1E). We also assessed the ability of these fungi to adhere to C20 and THP-1 cells by doing a short, 45-minute co-incubation prior to rigorous washing to remove non-adherent fungi (Fig. 1C). In this experiment, KN99α and H99 have decreased levels of adherence to C20 cells compared to BY4741, while there was no difference in THP-1 cells.

We then tested the ability of C20 cells to inhibit the growth of internalized KN99α and BY4741 (Fig. 1F). We saw that MG were not effective at controlling the growth of internalized KN99α at 24 or 48 h after all extracellular fungi were removed. Specifically, after 24 h, there were 12.74 times more KN99α colony-forming units (CFUs) than initially engulfed, and this increased to 68 times after 48 h. However, for BY4741, there was a decrease in CFUs after 24 and 48 h, with there being 0.76 and 0.44 times less fungi than initially engulfed, respectively. This indicates that C20 cells provide a permissive environment that promotes the intracellular survival and replication of *C. neoformans*; whereas, in contrast, they have fungicidal activity against BY4741. Furthermore, all experiments tested in Fig. 1 are done using serum-opsonized fungi, and we recognize that different opsonins could provide different results. Due to this, we tested if opsonization conditions, specifically serum, complement, and anti-capsule antibody, impact the phagocytosis and killing activity of MG (Fig. S1). While we saw some indications that opsonization can impact phagocytosis of BY4741, we saw no cryptococcal-specific differences relating to opsonization in engulfment or degradation by MG. Overall, our results indicate that both C20 cells and primary human MG have the same poor phagocytic activity for various *C. neoformans* strains, and these cells might recognize *C. neoformans* differently than THP-1 cells. Furthermore, the decreased phagocytic, adherence, and fungicidal activity seen with MG seems to be specific for *C. neoformans*.

### *C. neoformans* evasion of phagocytosis by microglia is not dependent on fungal viability, secreted factors, or capsule production

**Fig 2:**
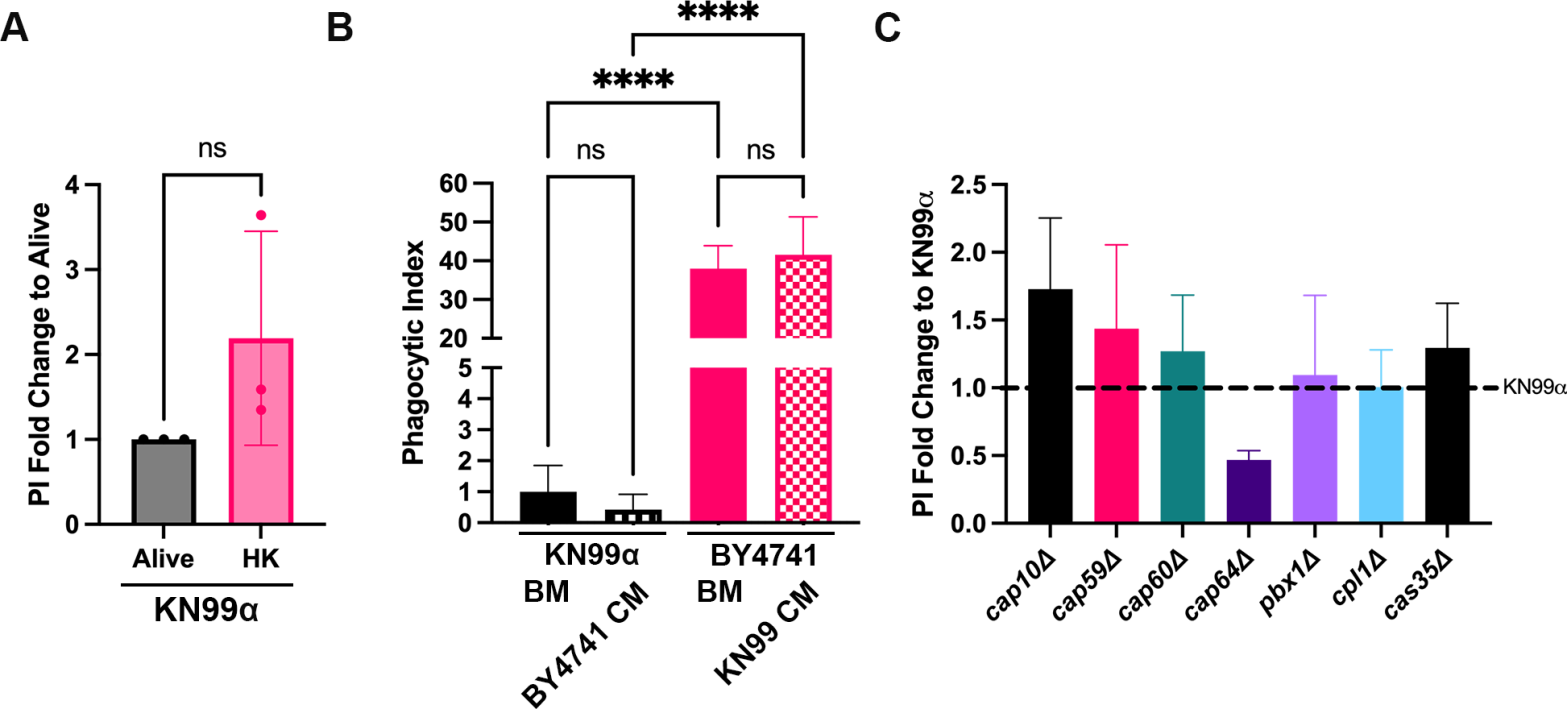
The ability of *C. neoformans* to be recognized and engulfed by C20 cells is independent of fungal viability, secreted factors, or capsule production/structure. (**A**) An uptake assay showing the ability of C20 cells to engulf live or heat-killed (HK) WT *C. neoformans* (KN99α). Fungi were heat-killed at 75°C for 30 min. The Y axis denotes the phagocytic index (PI) fold change relative to live KN99α. PI represents the number of internalized fungi per 100 microglia. Values represent the mean ± SEM from three biological replicates. Significance was determined using Welch’s t test, ns = not significant. (**B**) Uptake assay using conditioned media (CM) from fungal strains. CM was obtained by growing fungi for 72 h in 50% DMEM:Hams F-12, followed by centrifugation and filtration of the supernatant. Fungi were coincubated with C20 cells in either basal media (BM; 50% DMEM:Hams F-12) or the opposite fungi’s CM (i.e., KN99α incubated in BY4741-CM, and vice versa). Values represent the mean ± SEM from three biological replicates. Significance was determined using one-way ANOVA with multiple comparisons (Brown-Forsythe and Welch’s corrected), ****, *P* < 0.0001. (**C**) Uptake assay comparing the phagocytic index of capsular mutants to KN99α in C20 cells. Mutants were obtained from the Santiago-Tirado lab personal deletion library and the Madhani deletion collection. Y axis denotes the PI fold change to WT. Significance was determined using one-way ANOVA with multiple comparisons (Brown-Forsythe and Welch’s corrected). None of the strains tested were significant.

We next sought to identify factors that could be contributing to the ability of *C. neoformans* to evade phagocytosis by MG. First, we tested the ability of C20 cells to engulf live versus heat-killed KN99α (Fig. 2A). We found no statistically significant difference in the PI between viable (alive) or non-viable (heat-killed) *C. neoformans*. Next, we looked at the ability of secreted factors, present in conditioned media (CM), to inhibit phagocytosis by C20 cells. To do this, we prepared KN99α and BY4741 CM and added the BY4741-CM to live KN99α, and KN99α-CM to live BY4741 (in place of basal media, BM, 50% DMEM:Hams F-12) during co-incubation with C20 cells. If there were secreted cryptococcal metabolites, proteins, or capsular material that could inhibit phagocytosis by microglia, we would expect to see a decrease in phagocytosis of BY4741 when incubated with KN99α-CM. However, we saw that adding CM to either KN99α or BY4741 did not alter their PI (Fig. 2B). Specifically, when adding BY4741-CM to KN99α, the PI decreased from 1 to 0.43, with this decrease not being statistically significant. Similarly, adding KN99α-CM to BY4741 increased the PI slightly from 38 to 42, with this increase having no statistical significance. Finally, we looked at the impact of capsule production/structure on cryptococcal engulfment by MG. The polysaccharide capsule of *C. neoformans* is the yeast’s main virulence factor and has been extensively described as antiphagocytic, especially with regard to cryptococcal-macrophage interactions (31–33). Furthermore, the capsule and its main component (GXM) have been implicated in contributing to the pathogenesis of CME (34–36). Thus, we used various mutants that are defective in capsule organization, structure, or production and determined the ability of these strains to be phagocytosed by C20 cells. Surprisingly, we found that these mutants have no significant difference in phagocytosis by C20 cells compared to WT (Fig. 2C). Notably, *cap59*Δ, which lacks capsule production altogether, only had a PI 1.4 times that of KN99α. Overall, this data suggests that components other than secreted factors, capsule, or viability are responsible for *C. neoformans*’ ability to evade engulfment by MG.

### Microglia recognize *C. neoformans* differently than peripheral macrophages

**Fig 3:**
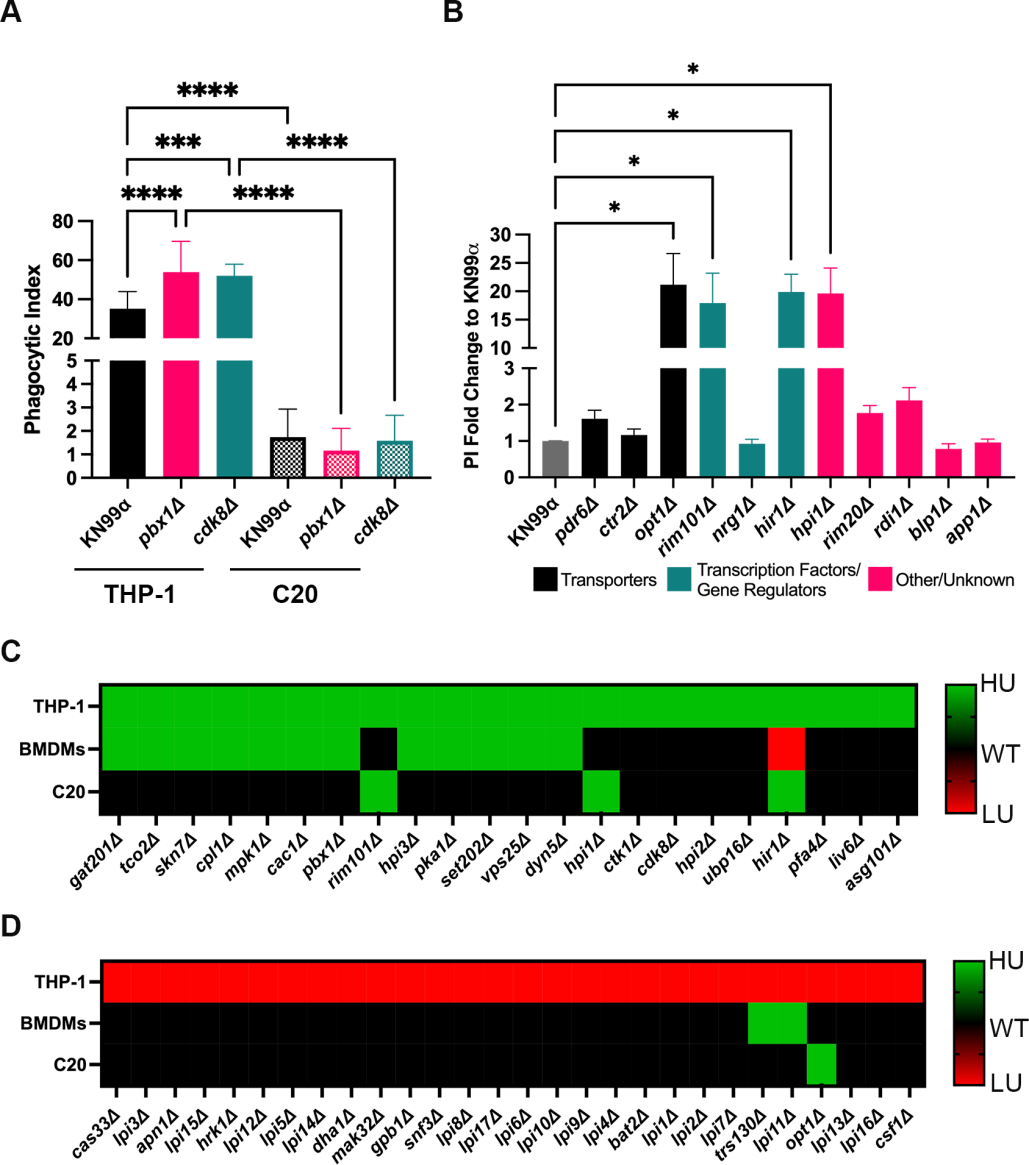
*C. neoformans* recognition and engulfment are distinct between microglia and other macrophages. (**A**) Uptake assay showing the ability of THP-1 cells and immortalized human microglia (**C20**) to engulf WT and mutant strains of *C. neoformans*. The mutants tested were characterized by Santiago-Tirado et al. (43) as having increased internalization by THP-1 cells and were obtained from the Madhani deletion collection. Phagocytic index (PI) represents the number of internalized fungi per 100 macrophages. Values represent the mean ± SEM from three biological replicates. Significance was determined using one-way ANOVA with multiple comparisons (Brown-Forsythe and Welch’s corrected), ***, *P* < 0.0005; ****, *P* < 0.0001. (**B**) Uptake assay showing the PI as fold change to WT of different cryptococcal mutants when coincubated with C20 cells. The mutants tested were described in previous literature as having increased internalization or altered interactions with macrophages. The mutants were obtained from the Madhani deletion collection. Values represent the mean ± SEM from three biological replicates. Significance was determined using one-way ANOVA with multiple comparisons (Brown-Forsythe and Welch’s corrected), *, *P* < 0.05. (**C, D**) Comparison of uptake assays done with C20 cells and various cryptococcal mutants with prior results with THP-1 cells and BMDMs. The results for each mutant in C20 cells were compared to the previously published results of these mutants from screens done with THP-1 and bone marrow-derived macrophages (BMDMs). These mutants were chosen because they were previously identified as those with increased (**C**) and decreased (**D**) internalization by our laboratory (43). Green represents mutants with increased internalization by the specified macrophage; red represents mutants with decreased internalization; and black represents mutants with no significant altered interactions with macrophages when compared to WT (the heat map used for comparison used the thresholds set forth by the various authors of the publications chosen). For the C20 screen, results indicate values that represented the mean ± SEM from three biological replicates (the values for some of the mutants with enhanced internalization by human microglia are shown in panel **B**). Significance was determined using one-way ANOVA with multiple comparisons (Brown-Forsythe and Welch’s corrected), *, *P* < 0.05.

To identify cryptococcal proteins that could contribute to the evasion of phagocytosis by microglia, we assessed the ability of C20 cells to engulf *C. neoformans* mutants that have shown increased engulfment by or altered survival within peripheral macrophages (37–44). As expected, two of these mutants, *pbx1*Δ (PI = 53.9) and *cdk8*Δ (PI = 52), show increased internalization by THP-1 cells compared to WT (PI = 35.1); however, these mutants show no difference in their phagocytosis levels by C20 cells (PIs of 1.7, 1.2, 1.6 for WT, *pbx1*Δ and *cdk8*Δ, respectively; Fig. 3A). Furthermore, a variety of mutants reported to be high uptake (HU) (such as *pdr6*D, *ctr2*D, *nrg1*D, *rim20*D, *rdi1*D, *blp1*Δ, and *app1*Δ) also showed no differences in their ability to be recognized and engulfed by C20 cells (Fig. 3B). To do a more extensive screen to identify antiphagocytic proteins relevant to microglia-cryptococcal interactions, we tested 50 of 56 mutants identified by Santiago-Tirado et al. (43) with altered internalization by THP-1 cells (we were unable to recover the remaining six from the frozen stocks). The 50 mutants were only tested in C20 cells, with comparison of our results (in C20 cells) to those published by Santiago-Tirado et al. (THP-1/AMs) and Gaylord et al. (bone marrow-derived macrophages; BMDMs) (39, 43). Almost all of the 50 mutants exhibited a different phenotype in C20 relative to THP-1 cells (Fig. 3C and D). In Santiago-Tirado et al. (43), mutants with significantly altered internalization by THP-1 cells were determined by having a PI two standard deviations away from the WT mean. In Gaylord et al., they determined hits with altered internalization by BMDMs by comparing uptake scores (PI fold change to WT) to the median uptake score for each plate of the deletion collection, then used permissive cutoffs to identify 10% of the strains tested as hits. For the C20 screen described in this paper, we tested nine mutants alongside WT (six technical replicates for each) at a time for one biological replicate, then repeated this for at least three biological replicates for all 50 mutants. The average PI values of each biological replicate (including mutants and WT) were compiled, and one-way ANOVA with multiple comparisons to WT was performed to assess significance between the 50 mutants. Since we only screened these mutants in C20 cells, all comparisons are using different thresholds for “increased” or “decreased” internalization. Thus, numerical values of significance cannot be compared, so the results are shown as a heat map of mutants with altered internalization as determined by the respective thresholds set forth by each author (this publication, Santiago-Tirado et al., and Gaylord et al.). Of the 22 mutants tested with increased internalization by THP-1 cells (green; HU), 12 of these also show increased internalization by BMDMs (Fig. 3C). In contrast, only three mutants that show increased internalization by THP-1 cells shared this antiphagocytic property with C20s (*rim101*Δ, *hpi1*Δ, *hir1*Δ; Fig. 3B and C). Of the 28 mutants tested with decreased internalization by THP-1 cells (red; low uptake [LU]), none resulted in decreased internalization by BMDMs or C20 cells (Fig. 3D). Surprisingly, three of these showed the opposite phenotype relative to THP-1 cells: *trs130*Δ and *lpi1*Δ were HU in BMDMs, and *opt1*Δ was HU in C20 cells (Fig. 3D). Overall, there were significantly more similarities between THP-1 and BMDMs than between either of them and C20s. Taken together, this indicates that MG recognize *C. neoformans* differently than peripheral macrophages, and this difference is exacerbated in mutants with increased internalization by peripheral macrophages.

### *C. neoformans* disrupts phagosome maturation in human microglia

**Fig 4:**
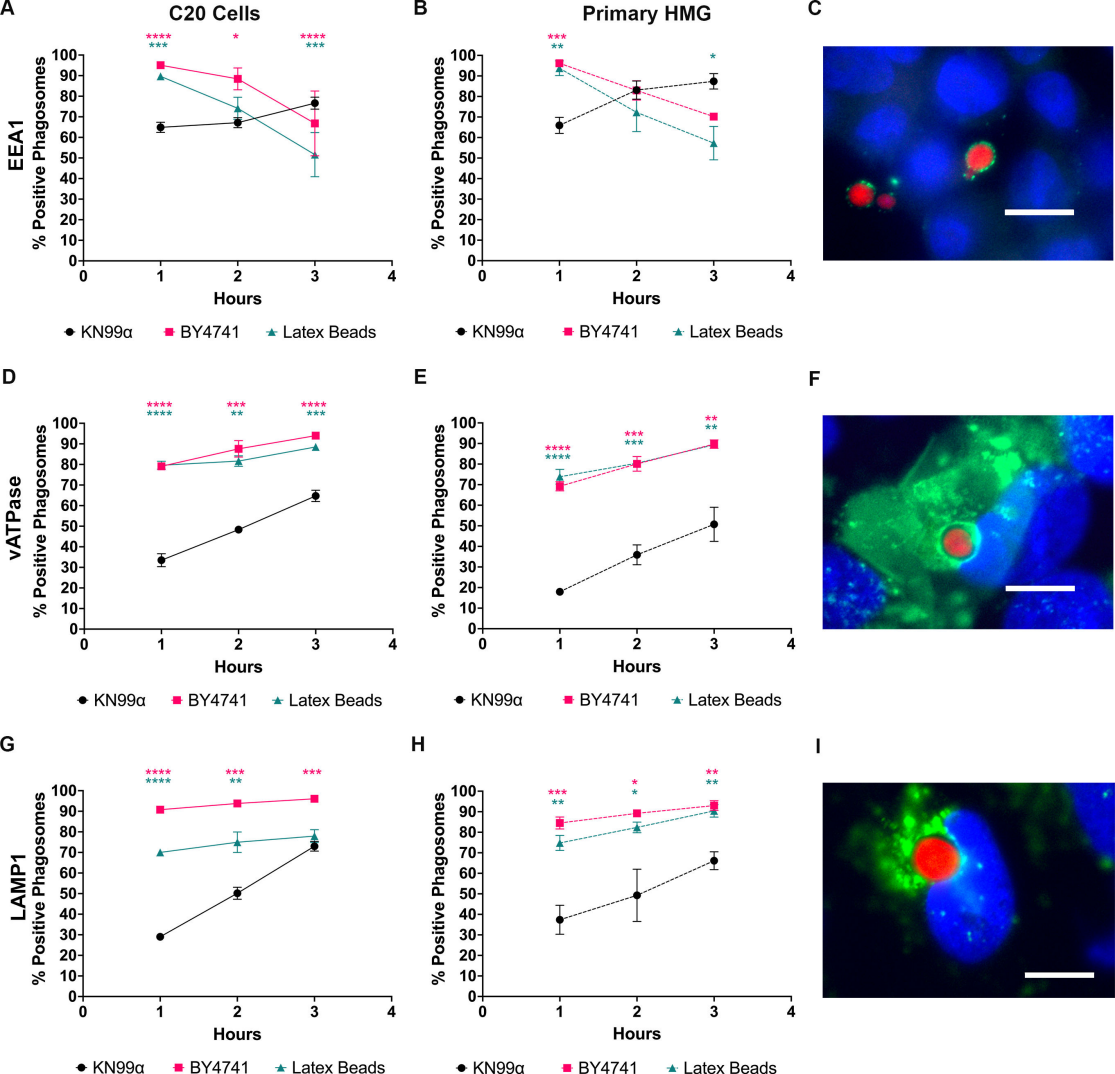
Maturation of cryptococcal phagosomes in immortalized and primary human microglia is altered. (**A, B**) Immunofluorescence showing the cargo-containing phagosomes that are positive for EEA1 association at 1, 2, and 3 h post-coculture with C20 cells (**A**) or primary human microglia (**B**). mCherry-expressing fungi or fluorescent latex beads were opsonized and incubated with microglia at an MOI of 20:1 (fungi) or 10:1 (latex beads) for 1, 2, or 3 h. Coverslips were fixed, stained with primary and secondary antibodies, and imaged via fluorescent microscopy. (**D, E**) Plotted are the cargo-containing phagosomes that are positive for vATPase association at 1, 2, and 3 h post-coculture with C20 cells (**D**) or primary human microglia (**E**). (**G, H**) Plotted are the cargo-containing phagosomes that are positive for LAMP1 association at 1, 2, and 3 h post-coculture with C20 cells (**G**) or primary human microglia (**H**). Merged representative images for cryptococcal-containing phagosomes positive for EEA1 (**C**), vATPase (**F**), and LAMP-1 (**I**). These images were taken at 100×, with blue showing the nuclei of the C20 cells, green showing the phagosomal marker, and red showing intracellular KN99α. The scale bar is 10 μm. Intracellular cargo shown are WT *C. neoformans* (KN99α) and *S. cerevisiae* (BY4741) and 5 um red fluorescent latex beads (CD Bioparticles) as controls. Lines representing C20 cells are solid, and dashed lines represent primary human microglia. Values represent the mean ± SEM from three biological replicates. *N* ≥ 50 phagosomes quantified per biological replicate. Significance was determined using one-way ANOVA with multiple comparisons (Brown-Forsythe and Welch’s corrected) for each time point, *, *P* < 0.05; **, *P* < 0.005; ***, *P* < 0.0005; ****P < 0.0001.

It has been shown in various types of peripheral macrophages that *C. neoformans* manipulates the phagosome maturation process by delaying association of maturation markers (17, 22, 23). Since we show that MG have altered interactions with *C. neoformans* relative to peripheral macrophages, we wanted to study the phagosome maturation process of KN99α in human MG after engulfment (Fig. 4). To do this, we performed immunofluorescence to assess the percentage of cargo-containing phagosomes positive for phagosome maturation markers (EEA1, LAMP1, and vATPase) after 1, 2, and 3 h of incubation with either C20 cells or primary human MG. Representative merged images showing positive association of all markers to cryptococcal-containing phagosomes are shown in Fig. 4C, F and I. In these images, blue is the nuclei of the C20 cells, green is the respective phagosomal marker, and red is the yeast. Individual channels for each image and additional representative images for the markers are shown in Fig. S2.

EEA1 is a marker for early phagosomes, and we found that our controls, BY4741 and 5 μm latex beads, had significantly more phagosomes with EEA1 association at earlier time points in both C20 cells and primary human MG than KN99α (Fig. 4A through C). Furthermore, EEA1 association follows the same trend for BY4741 and latex beads, with the number of percent-positive EEA1-associated phagosomes decreasing over time in C20 cells and primary human MG (from 95.1 and 89.7% to 66.8 and 51.6%, respectively, in C20 cells, and from 96.2 and 93.8% to 70.2 and 57.3%, respectively, in primary MG). However, in C20 cells and primary human MG, the percentage of EEA1-associated KN99α-containing phagosomes increases during the time course (from 64.9% to 76.6% in C20 cells and 65.9% and 87.4% in primary human MG). This indicates that KN99α not only delays EEA1 association to its phagosome but also retains it in both immortalized and primary human microglia.

vATPase accumulates on phagosomes as they progress through the maturation pathway and is the primary enzyme responsible for phagosome acidification. In C20 cells and primary human MG, all cargo-containing phagosomes increase in vATPase association during the 3-hour time course; however, KN99α has a significant decrease in vATPase-positive phagosomes at all time points compared to BY4741 and latex beads (Fig. 4D through F). Specifically, at 3 h in C20 cells and primary human MG, the percentages of KN99α-containing phagosomes positive for vATPase were 64.7 and 50.8, respectively, compared to BY4741-containing phagosomes, which were 94 and 89.7, respectively. This pattern is also seen with cargo-containing phagosomes positive for LAMP1 association. LAMP1 is a marker for late-stage phagosomes and lysosomes and is routinely used as a marker for phagolysosomes. In both types of microglia, all cargo-containing phagosomes increase in LAMP1 association over time (Fig. 4G through I). However, in both cell types, KN99α-containing phagosomes have significantly lower LAMP1 association than BY4741 and latex bead phagosomes at all time points (Fig. 4G through I). We also assessed the ability of different opsonins to impact the maturation of cargo-containing phagosomes (Fig. S1). While we saw trends that show unopsonized cargo has delayed phagosome maturation compared to their opsonized counterparts, we saw no cryptococcal-specific differences compared to the BY4741 and latex bead controls. Taken together, this data indicates that KN99α-containing phagosomes have delayed EEA1, LAMP1, and vATPase association, indicating altered phagosome maturation in immortalized and primary human MG. The similar trends for all phagosome maturation markers across MG cell types are also an indicator of the translatability of the C20 cell line to that of primary cells, especially as it relates to studying cryptococcal infection.

### *C. neoformans* induces phagosome membrane damage in human microglia

**Fig 5:**
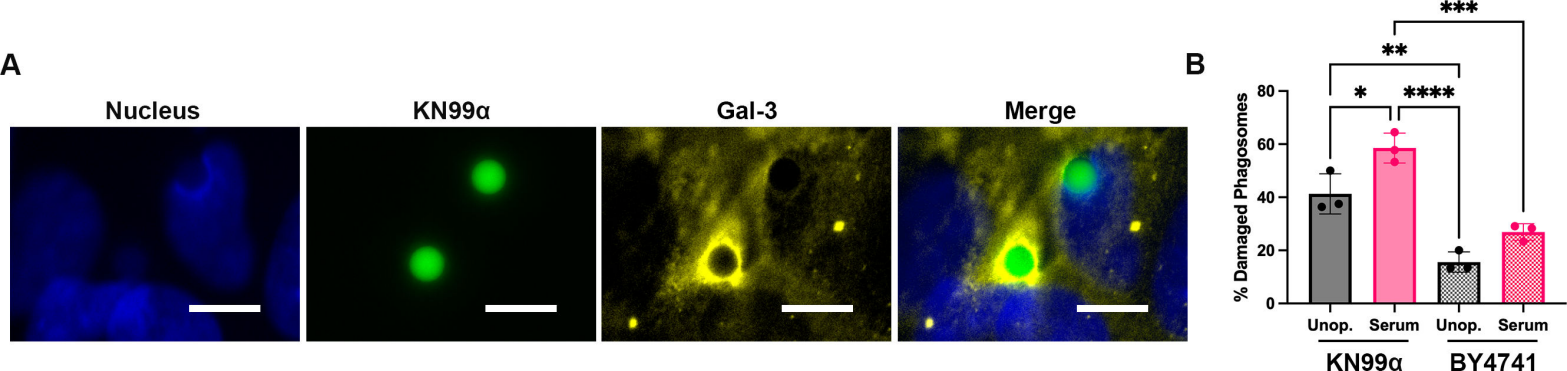
Phagosome membrane damage occurs in cryptococcal-containing phagosomes. (**A**) Representative image showing a cryptococcal-containing phagosome positive for Gal-3 association. Gal-3 is widely used as a reporter of phagosomal membrane damage. These images were taken at 100×, with blue showing the nuclei of the C20 cells, green showing intracellular KN99α, and yellow showing Gal-3. The scale bar is 10 μm. (**B**) Percentage of fungal-containing phagosomes positive for Gal-3 association 24 h after engulfment was analyzed by immunofluorescence as shown in panel **A**. Both *C. neoformans* (KN99α)- and *S. cerevisiae* (BY4741)-containing phagosomes were tested for membrane damage. mCherry-expressing fungi were either unopsonized (unop.) or opsonized (serum) and incubated with microglia at an MOI of 20:1 for 3 h. Wells were washed three times with DPBS to remove extracellular fungi, and microglia were incubated for an additional 24 h then assessed for membrane damage via Gal-3 staining. Values represent the mean ± SEM from three biological replicates. For all experiments, significance was determined using one-way ANOVA with multiple comparisons (Brown-Forsythe and Welch’s corrected), *, *P* < 0.05; **, *P* < 0.005; ***, *P* < 0.0005; ****, *P* < 0.0001.

Given that *C. neoformans* alters phagosome maturation in human MG, we wanted to test if the fungus also induces phagosome membrane damage, as this can lead to neutralization of the cryptococcal-containing phagosome. Galectin-3 (Gal-3) staining is widely used as a reporter of lysosomal membrane damage because it binds sugars on the luminal side of phagosomes only when membrane integrity is lost (45, 46). Using Gal-3 staining, we have shown that *C. neoformans* induces phagosome membrane damage in THP-1 cells as early as 4 h post-infection (22). Thus, we wanted to determine if *C. neoformans* also induces phagosome membrane damage in microglia. To do this, we incubated *C. neoformans* or *S. cerevisiae* with C20 cells for 3 h to allow for fungal engulfment. Extracellular fungi were washed, and the infected MG were incubated for an additional 24 h, and their phagosomes were assessed for membrane damage via immunofluorescence using an antibody against Gal-3. A representative image for positive association of Gal-3 to a cryptococcal-containing phagosome is shown in Fig. 5A. We found that both unopsonized and serum-opsonized KN99α and BY4741 had phagosomes positive for Gal-3 after 24 h (Fig. 5B). However, KN99α had significantly more Gal-3-positive phagosomes compared to BY4741, with an average of 41.3% and 15.6%, respectively, for unopsonized fungi. For serum-opsonized fungi, KN99α had 58.5% of phagosomes positive for Gal-3 compared to 26.9% for BY4741. Furthermore, serum-opsonized KN99α-containing phagosomes had significantly more Gal-3 association than phagosomes containing unopsonized KN99α (58.5% and 41.3%, respectively). Overall, this data indicates that MG are extremely sensitive to phagosome permeabilization by *C. neoformans* at 24 h after engulfment.

### Different cryptococcal strains alter phagosome maturation in microglia

**Fig 6:**
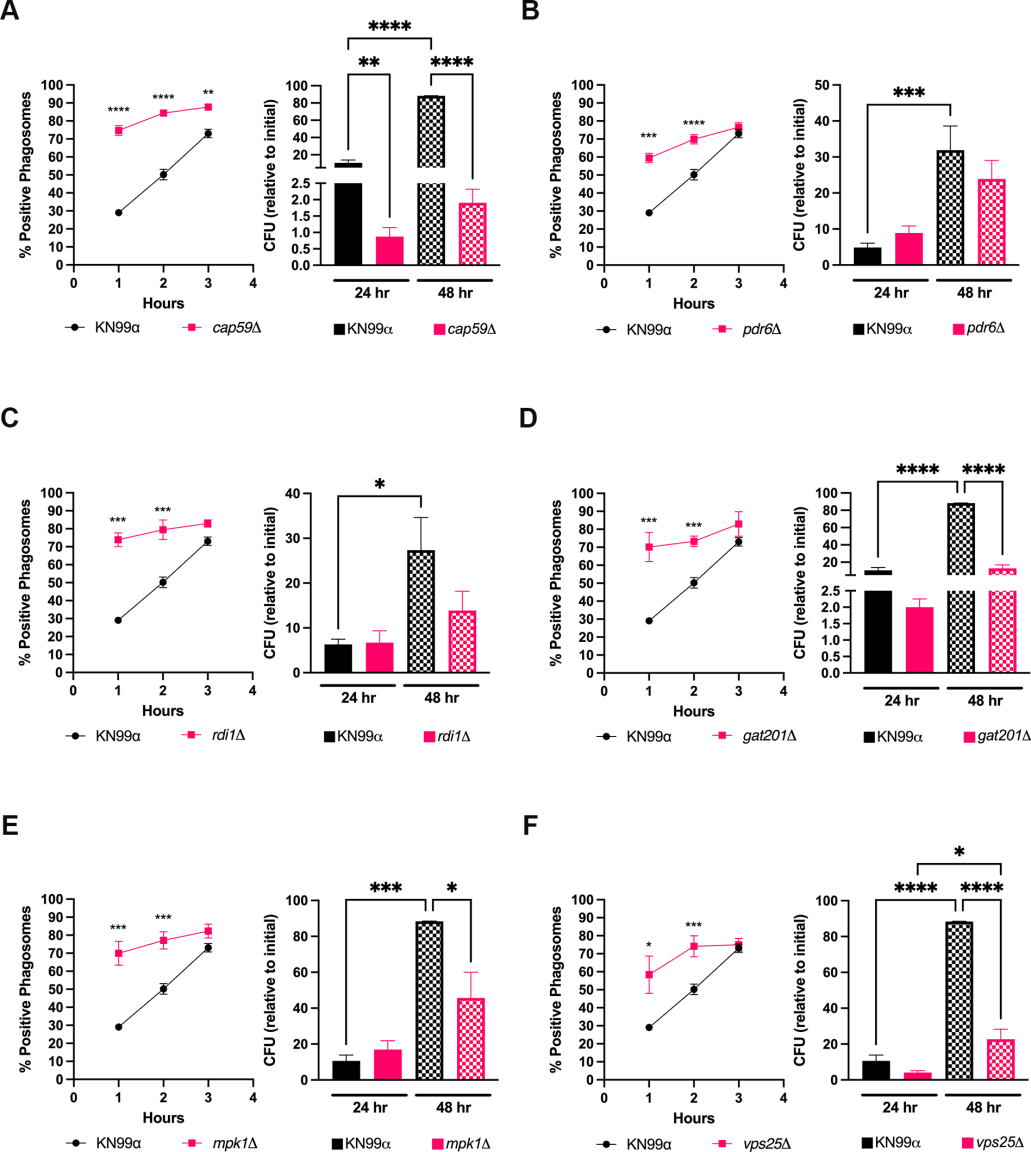
Phagosome maturation alterations in microglia do not completely predict fungicidal activity. (**A**) Immunofluorescence showing the KN99α- and *cap59*Δ-containing phagosomes that are positive for LAMP1 association at 1, 2, and 3 h post-coculture with C20 cells (left). *In vitro* survival assay assessing internalized KN99α and *cap59*Δ after 24 and 48 h post-engulfment by C20 cells (right). Shown are the colony-forming units (CFUs) that were obtained at each time point, normalized to the CFUs of initial engulfment (3 h). (**B–F**) Representation of experiments performed in panel A, however, with KN99α and *pdr6*Δ (**B**), *rdi1*Δ (**C**), *gat201*Δ (**D**), *mpk1*Δ (**E**), and *vps25*Δ (**F**). The mutants chosen for this figure all have been characterized as having altered survival within macrophages. Values represent the mean ± SEM from three biological replicates. For all experiments, significance was determined using one-way ANOVA with multiple comparisons (Brown-Forsythe and Welch’s corrected), *, *P* < 0.05; **, *P* < 0.005; ***, *P* < 0.0005; ****, *P* < 0.0001.

Since we show that KN99α alters phagosome maturation in both immortalized and primary human MG, we wanted to test the same with mutants with known altered *in vitro* and/or *in vivo* survival in peripheral macrophages (Fig. 6). *Cap59*Δ lacks capsule production and has been shown to be avirulent in various animal models (47–49). At all time points, *cap59*Δ has a significantly higher proportion of LAMP1-positive phagosomes compared to KN99α, indicating that this mutant has normal progression through the phagosome maturation pathway (Fig. 6A). Additionally, *cap59*Δ has significantly lower CFUs after 24 and 48 h post-engulfment compared to KN99α (Fig. 6A), reflecting its inability to survive intracellularly and supporting that C20 cells have enhanced fungistatic activity against this mutant. *Pdr6*Δ is a mutant that has been shown to have altered survival within macrophages and a mouse model (44, 50). In C20 cells, this mutant has increased LAMP1 association with phagosomes (albeit still at a lower percentage than controls) at 1 and 2 h compared to KN99α; however, this accelerated LAMP1 association did not lead to a significant reduction in normalized CFU count after 24 or 48 h post-engulfment (Fig. 6B). *Rdi1*Δ has difficulty surviving and proliferating within peripheral macrophage phagosomes and has attenuated virulence in a mouse model (42). We find that *rdi1*Δ has similar LAMP1 association and intracellular survival compared to *pdr6*Δ, with significantly higher LAMP1-positive phagosomes at 1 and 2 h compared to WT, and no significant reduction in normalized CFU count after 24 or 48 h post-engulfment (Fig. 6C). In a mouse infection model, *gat201*Δ is undetectable in lungs by 10 dpi, indicating fungal clearance (37). We find that *gat201*Δ has significantly increased LAMP1-positive phagosomes at 1 and 2 h and significantly reduced internalized CFU counts at 48 h (Fig. 6D). *Mpk1*Δ has attenuated virulence in a mouse model (51), and similar to the other mutants tested, *mpk1*Δ has accelerated LAMP1 association to its phagosomes compared to WT at 1 and 2 h and reduced internalized CFU after 48 h, indicating enhanced fungistatic activity of C20 cells (Fig. 6E). Lastly, *VPS25* encodes a member of the ESCRT complex, and its mutant (as well as mutants missing other proteins within this complex) has been shown to have altered survival within macrophages and attenuated virulence in mouse models (52). Following a similar trend to *gat201*Δ and *mpk1*D, *vps25*Δ has accelerated LAMP1 association to its phagosomes at 1 and 2 h and has significantly decreased normalized CFUs at 48 h post-engulfment by C20 cells (Fig. 6F). This indicates that C20 cells have enhanced fungistatic activity against *gat201*D*, mpk1*Δ, and *vps25*Δ. Taken together, all of the mutants tested have accelerated LAMP1 association to their phagosomes shortly after engulfment by C20 cells; however, this does not predict the fungicidal activity of microglia.

### Cryptococcal strains with increased internalization by microglia exhibit altered cell body size and cell wall composition

**Fig 7:**
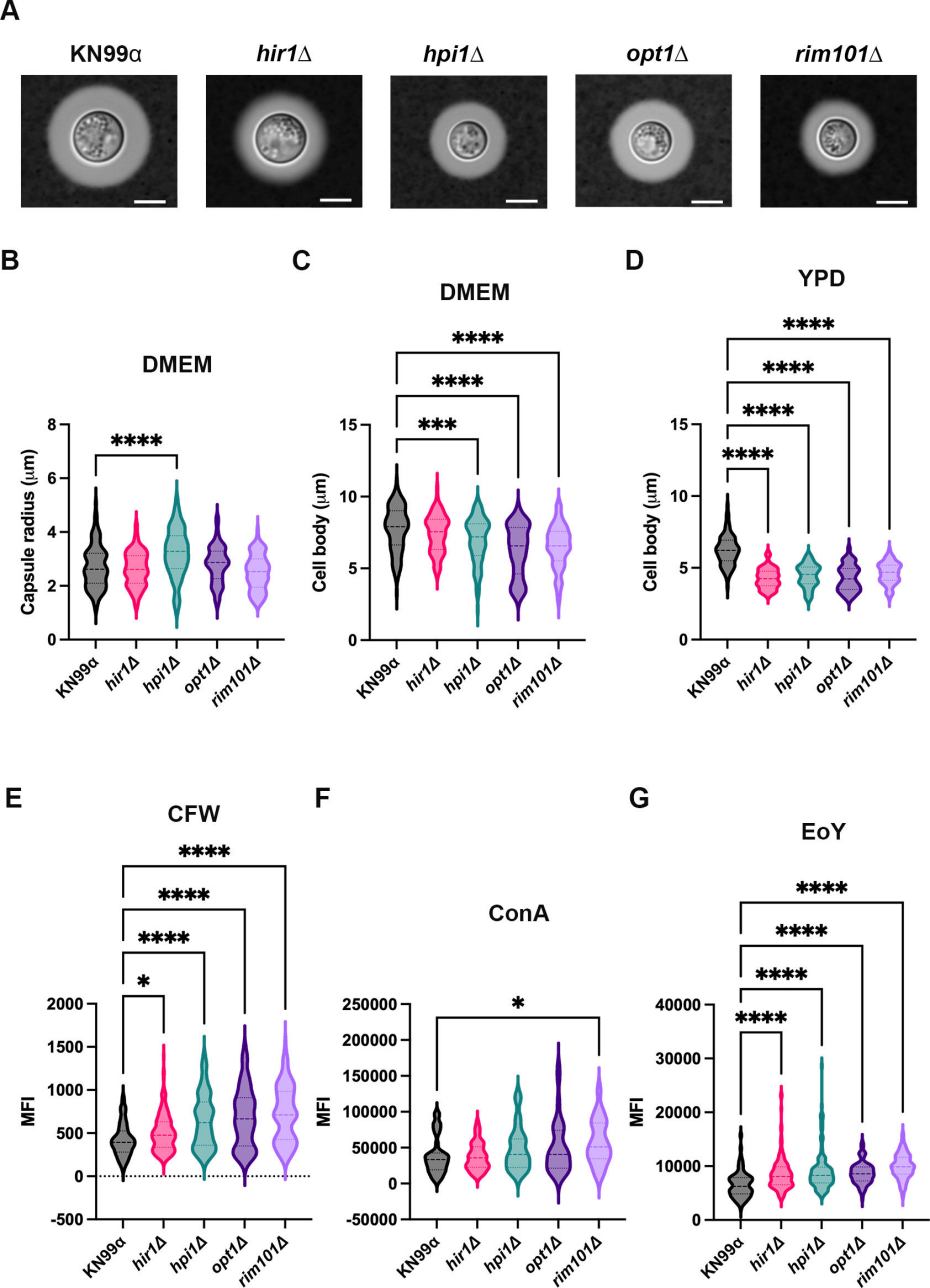
Cryptococcal cell body size and cell wall composition contribute to recognition and engulfment by microglia. (**A**) Representative capsule induction images of WT *C. neoformans* (KN99α) and the four mutants identified as having increased internalization by C20 cells in Fig. 3 (*hir1*D, *hpi1*D, *opt1*Δ, and *rim101*Δ). Capsules were induced by growing the fungal strains (from initial YPD overnight cultures) in DMEM for 24 h and imaged at 100× after India ink staining. The light gray shading around the cell body is the polysaccharide capsule. Scale bar represents 5 μm. (**B**) Quantification of the capsule radius in μm from the images represented in panel A. Capsule radius analysis determined via ImageJ software. Equation for capsule radius is: (total cell size including capsule − cell body size)/2. (**C**) Quantification of the cell body size in μm from the images represented in panel A. Cells initially grown in a YPD overnight culture were subsequently normalized and grown in DMEM for 24 h. Measurements taken using ImageJ software. (**D**) Quantification of the cell body size in μm from cells grown in liquid YPD for 18 h. Measurements taken using ImageJ software. (**B–D**) Values are represented as a violin plot showing all measurements (100 cells) from three biological replicates. (**E–G**) Quantification of mean fluorescent intensity (MFI) of fungal cells stained with calcofluor white (CFW; **E**), concanavalin A (ConA; **F**), or eosin Y (EoY; **G**). Fungal YPD overnight cultures were washed with PBS and stained with their respective cell wall dyes for 30 min. Cells were washed three times with PBS and imaged via fluorescent microscopy. Quantification of MFI of 100 cells was determined via CellProfiler pipeline. Values represent the mean ± SD from three biological replicates. For all experiments, significance was determined using one-way ANOVA with multiple comparisons (Brown-Forsythe and Welch’s corrected), *, *P* < 0.05; ***, *P* < 0.0005; ****, *P* < 0.0001.

Thus far, we have shown that *C. neoformans* evades phagocytosis by MG and, if internalized, can manipulate phagosome maturation, and that these processes can be altered by various mutants. Therefore, we wanted to study mutants that have increased engulfment by MG to identify factors that could be attributed to their altered recognition. We chose to study the four mutants identified as HU in Fig. 3 for variations in capsule production, cell body size, and cell wall structure (Fig. 7). We first assessed the ability of these mutants to produce a capsule (Fig. 7A and B). The only mutant that had altered capsule production compared to WT was *hpi1*Δ, which on average had a significantly larger capsule radius (Fig. 7B). We then assessed the cell body size of these mutants in host-like conditions (DMEM, 37°C, 5% CO_2_). All mutants, except for *hir1*Δ*,* had significantly smaller cell bodies compared to WT (Fig. 7C). Similarly, when assessing the cell body size of these mutants grown in nutrient-rich medium (YPD), all four mutants had significantly smaller cell bodies (Fig. 7D). Overall, this data indicates that cryptococcal mutants with increased internalization by MG generally have a smaller cell body size compared to WT. Lastly, we assessed the cell wall composition of the four mutants by staining with dyes known to bind specific components of the cell wall (Fig. 7E through G). All mutant strains had significantly increased staining of calcofluor white (CFW) and eosin Y (EoY), which are indicative of enhanced chitin and chitosan exposure, respectively. We also stained with concanavalin A (ConA) to assess mannoprotein exposure and found that only one mutant, *rim101*Δ, had significantly increased ConA staining compared to WT. Overall, this data indicates that enhanced chitin and chitosan exposure could contribute to enhanced recognition and engulfment by C20 cells.

### Assessment of additional virulence factors in mutants with enhanced engulfment by microglia

**Fig 8:**
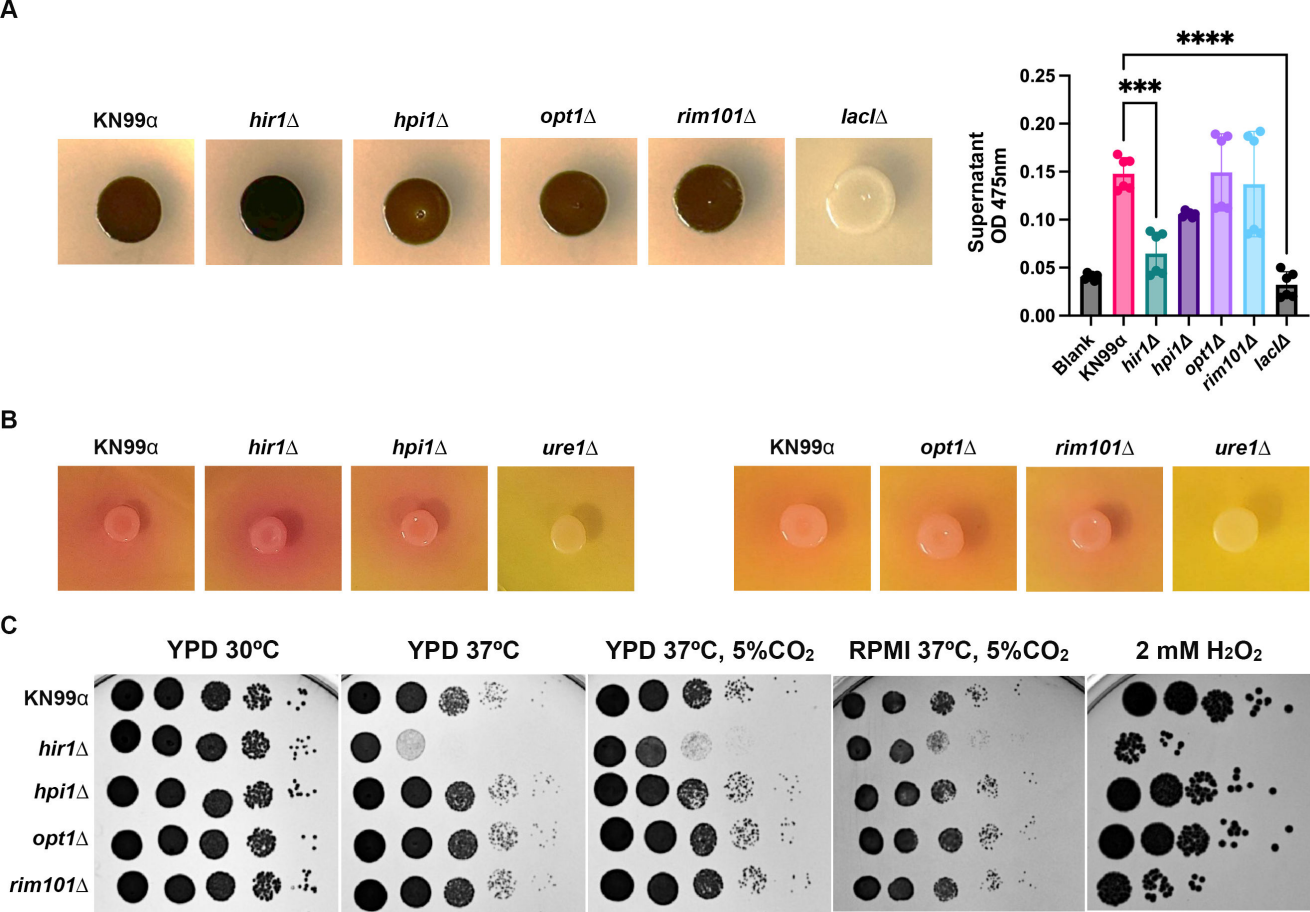
Assessment of stress phenotypes in mutants with high uptake by microglia. (**A**; left) Representative images of melanin production of WT *C. neoformans* (*neoformans* (KN99α) and the four mutants identified as having increased internalization by C20 cells in Fig. 3 (*hir1*D, *hpi1*D, *opt1*Δ, and *rim101*Δ). Fungal cultures grown in YPD were normalized to an OD_600_ of 0.25, spotted onto L-DOPA solid media, and incubated for 72 h. (**A**; right) Measurement of OD_475_ of supernatant of fungal cultures grown in melanin-inducing liquid media. L-DOPA fungal cultures normalized to an OD_600_ of 0.25 (from a YPD overnight culture) were grown for 18 h, pelleted, and the OD of the supernatant was measured. The liquid L-DOPA media induces melanization, and pelleting the 18 h cultures allows for visualization of melanin that has shed from the cryptococcal cell wall. A lower OD_475_ indicates less shed melanin. Blank was non-inoculated L-DOPA liquid media included as a control. *LacI*Δ is used as a negative control. Values represent the mean ± SD from three biological replicates. Significance was determined using one-way ANOVA with multiple comparisons (Brown-Forsythe and Welch’s corrected), ***, *P* < 0.0005; ****, *P* < 0.0001. (**B**) Representative images of urease production of the cryptococcal strains. *Ure1*Δ is used as a negative control. Fungal cultures from a YPD overnight culture were normalized to an OD_600_ of 0.25, spotted on Christensen Urea Agar media, and incubated for 72 h. A yellow background indicates no urease production. As urease is produced, the background changes to a pink/red. (**C**) Growth of cryptococcal mutants was assessed under various conditions. Serial dilutions of fungal YPD cultures were prepared and plated on nutrient-rich media (yeast-peptone-dextrose; YPD) and incubated at 30°C, 37°C, or 37°C with 5% CO_2_; plated on RPMI agar and incubated at 37°C with 5% CO_2_; and plated on YPD supplemented with 2 mM H_2_O_2_ and incubated at 30°C. All plates were incubated for 48 h.

Since we already tested the ability of *hir1*D, *hpi1*D, *opt1*Δ, and *rim101*Δ to produce capsule, we wanted to perform experiments assessing additional virulence factors of *C. neoformans*. Specifically, we tested melanization, urease production, growth at high temperature, and resistance to oxidative stress (Fig. 8). First, we incubated the cryptococcal strains on melanin-inducing solid media and found that *hir1*Δ qualitatively produces more melanin than WT (Fig. 8A). All other mutants had similar melanin production to WT. *Lac1*Δ was used as a negative control, as this mutant lacks the gene responsible for melanin production. We also tested the ability of these strains to retain melanin in their cell wall. To do this, we grew the strains in melanin-inducing liquid media overnight and pelleted the cultures. We measured the OD_475_ of the supernatant to quantify shed melanin. Blanks (non-inoculated liquid L-DOPA media) and *lac1*Δ were used as controls for negative melanin production/shedding. Again, *hir1*Δ is the only mutant with a significant phenotype, exhibiting decreased shed melanin compared to WT. Overall, this data indicates that *hir1*Δ has cell wall alterations that contribute to less shed melanin. This finding may explain the qualitative “hypermelanization” phenotype seen with *hir1*Δ on solid medium: with less shed melanin, more melanin will accumulate on the cell wall, leading to a darker appearance on solid medium.

Next, we assessed urease production. To do this, we plated the strains on urea agar and incubated them for 5 days, including *ure1*Δ as a negative control for urease production (Fig. 8B). As the strains produce urease, the urea in the agar is degraded into ammonia, which increases the pH of the medium, leading to a visible color change. Overall, none of the mutants show altered urease production compared to WT.

Lastly, we tested the ability of the *C. neoformans* strains to grow under various stress conditions using agar plates (Fig. 8C). Under optimal growth conditions, such as YPD at 30°C, none of the mutants show a growth defect. However, on YPD at 37°C, *hir1*Δ exhibits a growth defect compared to WT. This defect is partially rescued with the addition of 5% CO_2_. Similarly, *hir1*Δ has a minor growth defect on RPMI media, which mimics host-like conditions. We also tested the ability of the strains to grow under oxidative stress, as this is one of the main stresses the fungus encounters inside a host. When growing these strains on agar plates containing 2 mM H_2_O_2_, we found that *hir1*Δ and *rim101*Δ have increased sensitivity to oxidative stress compared to WT. Overall, this data indicates that *hir1*Δ exhibits a growth defect at high temperature, and 2 of the four mutants have increased sensitivity to oxidative stress.

## DISCUSSION

Studies of cryptococcal-MG interactions have lagged behind those focusing on peripheral macrophages, such as AMs or BMDMs. Most of what we know regarding MG and *C. neoformans* comes from *in vivo* observations in various animal models of *C. neoformans* pathogenesis (15, 53, 54). However, in-depth *in vitro* interactions studying recognition, phagocytosis, and intracellular survival of *C. neoformans* by human MG remain widely understudied. Here, we present an initial characterization of cryptococcal-MG interactions, focusing on phagocytosis evasion, phagosome maturation, and recognition of cryptococcal mutants. We do this using C20 cells, which are a relatively recent and highly translational immortalized human MG cell line (26). We not only define initial cryptococcal-MG interactions, but also validate the use of C20 cells, when compared to primary human and murine MG, for studying these interactions *in vitro*.

We show that immortalized and primary human MG have similar levels of phagocytosis of WT *C. neoformans*, and we directly compare this with the phagocytic activity of THP-1 cells, a widely used model for AMs. While it has been implied that both human and murine MG have little to no phagocytic activity against *C. neoformans* (13, 55–57), we are the first to directly compare these phagocytes and show that MG are significantly defective in adherence to and internalization of *C. neoformans* relative to AMs. Furthermore, we find that these low levels of cryptococcal engulfment and adherence are not due to an inherent defect in phagocytosis, as C20 cells and primary murine MG have significantly higher internalization and adherence of *S. cerevisiae* compared to *C. neoformans*. We have used a variety of MG (i.e., primary human and murine) to validate the usage of C20 cells to study these fungal interactions; however, we do recognize that primary murine MG have higher levels of engulfment of WT *C. neoformans* compared to that of C20 cells and primary human microglia. This is not surprising, as many publications have highlighted the differences between rodent-derived and human MG, specifically noting their distinctions in RNA expression, immunophenotypes, and nitric oxide activity (28, 58–63). This finding does not diminish the credibility of C20 cells; rather, it highlights the translatability of this cell line, specifically when comparing it to primary human MG. We also show, like others have published, that *C. neoformans* survives and replicates inside immortalized human MG, suggesting that C20 cells act as a type of reservoir for cryptococcal infection, further showcasing the validity of using the C20 cell line.

Having shown that C20 cells behave just like primary MG in terms of cryptococcal interactions, we then tried to identify fungal factors that could be mediating these limited levels of engulfment by MG. While it has been shown that viability is needed for phagosome maturation manipulation by *C. neoformans* (18, 23), we were surprised to see that cryptococcal viability does not, however, impact recognition and engulfment by C20 cells. Furthermore, we find that secreted cryptococcal factors, such as shed capsular material, also do not contribute to phagocytosis evasion by microglia. Similarly, mutants either lacking capsule altogether (*cap59*Δ) or with altered capsule structure/production (*cap10*D, *cap60*D, *cap64*D, *pbx1*D, *cpl1*Δ, and *cas35*Δ) also are not engulfed by C20 cells to the same extent as non-pathogenic controls. GXM, the main component of the *C. neoformans* polysaccharide capsule, has been extensively described as antiphagocytic and plays a significant role during cryptococcal pathogenesis within the CNS (35, 64–66). However, GXM production and shedding are not the only antiphagocytic mechanisms employed by *C. neoformans*, as other mechanisms have been described, albeit only in peripheral macrophages (37, 67, 68). Thus, it is conceivable that a novel mechanism is at play in the CNS environment, specifically affecting microglia. Although we acknowledge that more testing is necessary, specifically following conditions that induce capsule production, since 3-hour coincubation with C20 cells in DMEM:Hams F-12 might not be sufficient to produce robust capsule and cause a change in phagocytic activity, we believe that C20 cells represent a powerful way to start addressing this question.

Given that C20 cells engulf *S. cerevisiae* cells robustly, we hypothesize that the MG antiphagocytic phenotype is mediated by cryptococcal proteins. To begin deciphering this, we tested mutants that have been previously characterized as having altered internalization by peripheral macrophages (THP-1 and BMDMs). We found that most of the mutants tested did not reflect the same phagocytic activity by C20 cells that was seen with peripheral macrophages. Notably, there was some concordance between the two peripheral macrophage cell types; however, it is important to highlight the limitations of these comparisons. First, the screens that were done in other macrophages had different thresholds for identifying mutants with altered internalization (these thresholds for THP-1 and BMDMs compared to C20 cells are stated in the Results section). Second, similar to differences between rodent-derived and human MG, murine-derived BMDMs and human-derived THP-1 cells have significant differences in their RNA expression, immunophenotypes, and metabolic activity (69–73). Third, while no studies have been done looking at specific differences between MG and AMs, many studies have looked at the differences in receptor expression and immune responses between MG and other macrophages within the CNS, including microglia-like cells, which are BMDMs that colonize the CNS under pathological conditions (74–79). For example, naïve MG have lower expression of CD45 and CD206 compared to peripheral macrophages, and they lack CD163, CD44, and CD169 expression altogether (80). Under activated conditions, MG have higher expression of CX3CR1 and IL1RL2 and lower expression of Clec4e (C-type lectin domain family 4), TLR-8, and CCR2 compared to infiltrating macrophages (75). Furthermore, it has been shown that different tissue-resident macrophages, such as Kupffer cells and AMs, have differences in their phagocytic and killing activity of *C. neoformans* (81, 82). Thus, we hypothesize that in addition to fungal factors mediating microglia-specific phagocytosis evasion, differences in immunophenotypes and phagocytic receptor expression between MG and peripheral macrophages are contributing to the defect seen in recognition and engulfment by C20 cells. Although more work is needed to elucidate the differences between these phagocytes, specifically regarding cryptococcal infection, our data is consistent with both fungal and host factors mediating this phenotype.

Consistent with a fungal contribution, we identified four mutants with increased internalization by C20 cells relative to WT. We sought to look for commonalities between these mutants that could explain their differential recognition and engulfment by microglia. We first looked at capsule production and found that none of the mutants had altered capsule production compared to WT, except for *hpi1*Δ, which had a significantly larger capsule radius. However, since the capsule is antiphagocytic due to its ability to hide the pathogen-associated molecular patterns (PAMPs) on the fungal cell surface, it is unlikely that an increase in capsule size compared to WT is a contributing factor to this mutant’s enhanced engulfment by C20 cells. However, when we looked at cell body size, overall, the mutants were smaller. In host-like media, we found that all mutants, except for *hir1*Δ, had significantly lower cell body size, and in nutrient-rich media, all mutants exhibited significantly lower cell body sizes compared to WT. This phenotype could directly contribute to the ability of these mutants to be engulfed by microglia, as it has been shown that smaller *C. neoformans* cells have increased association with liver macrophages (83). We also looked at the exposure of cryptococcal cell wall components of these mutants compared to WT and found that all mutants had significantly higher intensity of chitin (stained with CFW) and chitosan (stained with EoY) on their cell walls compared to WT. While we were not surprised to find that these mutants have alterations in their cell wall, we were surprised to find a unanimous cell wall phenotype for all the mutants with enhanced engulfment. This could indicate that MG has increased expression of receptors for chitin and chitosan, or for another PAMP exposed in all of these mutants. We also looked at the ability of these mutants to produce virulence factors, such as melanin and urease. We saw that, relative to WT, *hir1*Δ had significantly less shed melanin concomitant with melanin accumulation on the cell wall, and this could be a consequence of cell wall alterations that could potentially enhance recognition and engulfment by microglia. None of the other mutants had defects in melanin, and all of them showed normal urease production. Lastly, we tested the ability of these mutants to grow under a variety of stressors and found that *hir1*Δ has a significant growth defect at high temperature compared to WT, and it, along with *rim101*Δ, exhibits increased sensitivity to oxidative stress. Thus, the only commonality found was the overall smaller size of these mutants and the increased exposure of chitin and chitosan.

Lastly, we studied the maturation of WT cryptococcal phagosomes in C20 cells and primary human MG. Relatively little is known about phagosome maturation in MG after *C. neoformans* engulfment, despite several studies published about cryptococcal manipulation of phagosome maturation and acidification in peripheral macrophages (17, 23). We find that WT *C. neoformans* manipulates phagosome maturation in both C20 cells and primary human MG by retaining EEA1 in and delaying vATPase and LAMP1 recruitment to its phagosomal membrane. Since EEA1 is an early endosomal marker, it still remains unclear whether vATPase and LAMP1 association is perturbed in cryptococcal phagosomes due to delayed EEA1 association and retention after engulfment. Furthermore, it has been shown that peripheral macrophages accumulate LAMP1 in cryptococcal-containing phagosomes much faster than MG (17). Specifically, 60–70% of cryptococcal phagosomes are positive for LAMP1 just 30 min after coculture with BMDMs (17), compared to the 3 h it takes for cryptococcal phagosomes to reach this level of LAMP1 association after engulfment by MG. While we already show that MG respond differently to *C. neoformans* than macrophages, it is important to note that their findings resulted from the use of murine macrophages and antibody-opsonized H99. However, while more direct comparisons need to be done regarding MG vs macrophage cryptococcal phagosome maturation, we believe that MG have significant delays in recognition and phagosome maturation compared to other macrophages. Furthermore, while ~70% of cryptococcal phagosomes become positive for LAMP1 after 3 h post-coculture with C20 cells and primary human MG, this does not always correlate to fungicidal activity within C20 cells. We assessed phagosome maturation and fungicidal activity of C20 cells against cryptococcal mutants known to have altered intracellular or *in vivo* survival and found that although all mutants had increased association of LAMP1, some were still growing inside microglia. This indicates that alterations in the expression of PAMPs in the cryptococcal cell wall can have drastic effects not only on recognition and engulfment but also on phagosome maturation. Although studies have shown that both pathogens and host immune cells alter phagosome maturation based on PAMP exposure and subsequent pattern recognition receptor engagement (84), this is surprising for the cryptococcal field, where typically mutants that cannot alter phagosomal maturation are defective in intracellular growth (22). However, we do acknowledge that it was a small number of mutants, and more detailed studies are needed, such as evaluating phagosome acidification and phagolysosome function (i.e., cathepsin activity) in MG. It is clear that altered PAMP exposure and phagosome maturation do not necessarily correlate with enhanced fungicidal activity by C20 cells.

While many studies have looked at the accumulation of damage on cryptococcal phagosomes in peripheral macrophages, this had not been tested directly in MG. Notably, in THP-1 cells, only 5% to 10% of cryptococcal phagosomes are positive for Gal-3 after 24 h (22). This is in direct contrast to our results that show almost 60% of phagosomes are positive for Gal-3 staining in C20 cells after 24 h. Moreover, we have previously shown a good correlation between the percentage of damaged phagosomes and the percentage of cryptococcal-containing phagosomes with altered acidification (22); thus, we would expect a similar increase in phagosomal pH alterations in C20 cells. While these findings cannot be directly compared, this further supports our hypothesis that MG are ineffective against *C. neoformans* to an exacerbated extent compared to that of other macrophages. Also, we show that the opsonization status of the fungi has a significant effect on the membrane damage accumulation on cryptococcal phagosomes after 24 h. We believe this is due to the fact that opsonization activates different receptors on the microglial cell surface, resulting in different phagosome maturation dynamics (85–87). This highlights the importance of studying the roles of opsonization during cryptococcal infection in the brain. This is important given that the BBB becomes permeable during cryptococcal infection, meaning antibodies, which are not normally in the brain under steady-state conditions, could potentially enter the brain parenchyma and opsonize disseminated *C. neoformans*. Furthermore, initial exposure to *C. neoformans* can lead to low levels of circulating antibodies in the blood that could be used to opsonize the yeast later on during infection before dissemination to the brain.

In conclusion, we demonstrate the use of C20 cells to study cryptococcal-MG interactions and have found that MG recognize and respond to cryptococcal infection differently than that of peripheral macrophages. We also found that *C. neoformans* manipulates phagosome maturation in both immortalized and primary human MG, and this is dependent on capsule and cell wall modifications. Furthermore, we show that mutants with increased internalization by MG can be identified using the C20 cell line, and enhanced internalization could be attributed to a smaller cell body size and altered cell wall composition. Overall, we believe that C20 cells can be used to better define cryptococcal-MG interactions by allowing future mechanistic studies that can then be validated *in vivo*.

## MATERIALS AND METHODS

### Strains, cell lines, and growth conditions

Strains used were *C. neoformans* serotype A strains KN99α and H99 (88), *C. gattii* strain R265, *C. neoformans* serotype D strain Jec20 (89), and *S. cerevisiae* strain BY4741 (90) expressing mCherry (91). For some assays, the *C. neoformans* strain was expressing mCherry (92). The *C. neoformans* mutants were obtained from the Madhani deletion collection (40). All fungal strains were maintained at −80°C and grown at 30°C on yeast-peptone-dextrose (YPD) plates.

The human monocytic cell line THP-1 (ATCC TIB-202) was grown in THP-1 complete media (RPMI-1640 with L-glutamine supplemented with 1 mM sodium pyruvate, 0.05% β-mercaptoethanol, 10% heat-inactivated fetal bovine serum [FBS], and 1× Pen-Strep solution [100 units/mL penicillin and 100 μg/mL streptomycin]) and passed two times per week. The cells were never used after 12 passages. THP-1 cells are a monocyte-derived cell line that can serve as a model for AMs when differentiated using phorbol 12-myristate 12-acetate (PMA), which is widely accepted in the field (93–96). For differentiation into monocyte-derived macrophages, the THP-1 cells were treated as described (43), except we used 0.16 μM PMA (Millipore Sigma).

The human microglia cell line C20 (26) was grown in C20 complete media (DMEM-F12 with 10% FBS, 1× Pen-Strep solution [100 units/mL penicillin and 100 μg/mL streptomycin], and 1× N-2 supplement [Gibco #17502048]). Cells were supplemented separately with 1 mM fresh dexamethasone. C20 cells were passaged two to three times per week and were never used after 12 passages.

The primary human microglia cells were obtained from Celprogen (#37089-01) and grown in Human Microglia Primary Cell Culture Complete Media with Serum (Celprogen #M37089-01S). Cells were passaged three to four times per week and were never used after eight passages.

### Immunofluorescence

C20 cells were seeded at a density of 2.74 × 10^5^ cells/mL on collagen-coated coverslips (#1.5 round 12 mm glass coverslips, Electron Microscopy Sciences) that were incubated for 1 h at room temperature (RT) in 50 μg/mL rat-tail collagen IV (Corning) diluted in 0.02 N acetic acid and washed 3× with DPBS. Primary human microglia were seeded at a density of 2.74 × 10^5^ cells/mL on #1.5 round 12 mm pre-treated German glass coverslips (Electron Microscopy Sciences). The cells were activated overnight with 0.2 μg/mL TNF-α prior to challenge with fungal strains or latex beads. Fungal strains were grown overnight in YPD, harvested in the log phase (OD_600_ of 0.6–0.8), and washed 2× with PBS. Strains not expressing mCherry were incubated with Lucifer Yellow (250 μg/mL) for 30 min at 30°C with rotation and washed 2× with PBS. The cell counts were collected (Bio-Rad Cell Counter), and fungal cells were either unopsonized (PBS) or opsonized with 40% human serum (Sigma Aldrich) at a ratio of 1 × 10^5^ cells/µL of serum. For additional opsonization experiments, fungi were opsonized with 10% human complement (Pel-Freez Biologicals) or 10 μg/mL 18B7 antibody (a gift from Arturo Casadevall). Fungal cells were washed 1× in PBS, resuspended in prewarmed basal media (for microglia: DMEM-F12; for THP-1s: RPMI), and added to the microglia-adhered coverslips at an MOI of 20:1. In place of fungal strains, 5 μm fluorescent latex beads (CD Bioparticles) were washed 1× in PBS and treated as above. At specified time points, the wells containing the C20-*Cryptococcus*/beads were washed with DBPS, fixed with 3.7% formaldehyde in PBS for 10 min at RT, washed with PBS, and permeabilized with 0.3% saponin in PBS for 10 min at RT. Cells were washed with PBS and blocked for 30 min in 10% goat serum in PBS. Cells were immunolabeled in 1% goat serum at 4°C overnight with an antibody against Gal-3 (rat; M3/38: sc23938, Santa Cruz Biotechnology; 1:100 concentration), LAMP1 (mouse; Developmental Studies Hybridoma Bank, H4A3; 1:500 concentration), EEA1 (rabbit; Abcam, ab2900; 1:100 concentration), or vATPase (mouse; Santa Cruz Biotechnology, D-11; 1:500 concentration). Coverslips were washed with PBS, and a corresponding Alexa Fluor-conjugated goat secondary antibody (Invitrogen) was diluted to 1 μg/mL in PBS with 2 μg/mL of 4′6-diamidino-2-phenylindole (DAPI; Millipore Sigma) and added to cells for 1 h at RT. Cells were washed with PBS, and coverslips were mounted to imaging slides with 5 μL Prolong Diamond Antifade Mountant (Invitrogen) and allowed to cure for at least 24 h before imaging. Images (Z-stacks) of fixed cells were acquired using a Zeiss Axio Observer 7, with an Axiocam 506 mono camera and a 100×/1.4 plan-apo oil objective. Quantification of images was obtained using ImageJ by visualizing phagosome marker expression and its association to internalized fungi on the image files. Fifty phagosomes were quantified per condition per biological replicate. Percent positive phagosomes were determined by dividing the number of positive phagosomes by the number of total phagosomes quantified and multiplying by 100.

### Uptake assays

C20 cells/primary human microglia were activated with 0.2 μg/mL TNF-α and seeded on glass-bottom 96-well plates (Corning) at a density of 2.74 × 10^5^ cells/mL and allowed to adhere overnight. THP-1 cells were seeded at a density of 2.74 × 10^5^ cells/mL and differentiated with 0.16 μM PMA for 48 h, followed by a recovery step of 24 h without PMA. The microglia/macrophages were challenged with fungi as above. After 3 h, the cells were washed, fixed, and permeabilized as above. Cells were then stained with 2 μg/mL of DAPI and 2.5 μg/mL of CellMask Deep Red (Invitrogen) for 10 min at RT. Cells were washed with PBS and stored at 4°C in 1 mM NaN_3_ until imaging. The plates were imaged on a Zeiss Axio Observer microscope with an automatic stage. Each well was imaged using a 3 × 3 grid set up, and resulting images were analyzed using a CellProfiler (97) pipeline to determine the PI values.

For heat-killed fungi, *C. neoformans* YPD cultures were harvested in log phase, washed 2× with PBS, and incubated either at 75°C (heat-killed) or RT (alive) for 30 min. Cells were washed 1× in PBS, and cell counts were attained. For conditioned media, overnight fungal cultures in YPD were diluted to 1 × 10^5^ cells/mL in DMEM/F-12 and incubated for 72 h at 37°C with 5% CO_2_ in a T75 flask (VWR). Cells were removed by centrifugation, and the supernatant was filtered through a 0.22 μm filter. The conditioned media was used in place of DMEM-F-12 when preparing the MOI suspension.

### Adherence assay

C20 cells were activated with 0.2 μg/mL TNF-α and seeded on 24-well plates (one per timepoint) at a density of 2.74 × 10^5^ cells/mL and allowed to adhere overnight. THP-1 cells were seeded at a density of 2.74 × 10^5^ cells/mL and differentiated with 0.16 μM PMA for 48 h followed by a recovery step of 24 h without PMA. The macrophages were challenged with fungi as above. The inoculum for each fungus was diluted and plated on YPD. CFUs were counted after 2-day incubation at 30°C. Forty five minutes after challenge, plates were washed 2× with DPBS to remove extracellular fungi. Nine hundred microliters of killing buffer (0.1% Triton X-100, 1 mM EDTA) were added to each well, and the cells were lysed for 5 min at RT. Lysates were diluted in DPBS, spread on YPD, and CFUs were counted after 2-day incubation at 30°C. To determine percent adherence, the CFU # of adherent fungi (after 45 min challenge) was divided by the CFU # from the initial inoculum and multiplied by 100.

### Killing assays

C20 cells were activated with 0.2 μg/mL TNF-α and seeded on 24-well plates (one per timepoint) at a density of 3.34 × 10^5^ cells/mL and allowed to adhere overnight. C20 cells were challenged with fungi as above. Three hours after challenge, plates of all time points were washed 2× with DPBS to remove extracellular fungi. The 24- and 48-hour plates were fed with DMEM-F-12 and returned to the incubator for their specified time points (the 48-hour plate was washed 2× with DPBS after an additional 24-hour incubation and fed with DMEM-F-12). At the specified time points after DPBS washes, 900 μL of killing buffer (0.1% Triton X-100, 1 mM EDTA) was added to each well, and the cells were lysed for 5 min at RT. Lysates were diluted, spread on YPD, and CFUs were counted after 2-day incubation at 30°C.

### Isolation of primary murine microglia

Microglia were isolated from C57BL/6 via immunopanning as described (98, 99). Briefly, 6 μg/mL of goat anti-rat IgG secondary antibody (Invitrogen) is diluted in 50 mM Tris-HCl (pH 9.5) and added to a 15 mm cell culture dish to prepare the panning plate. The panning plate is incubated at 37°C with 5% CO_2_ for 3 h. The panning dish is rinsed 3× with panning buffer (0.2% BSA in DPBS), and 1 μg/mL of rat anti-mouse CD11b antibody (Invitrogen; M1/70) diluted in panning buffer is added to the panning plate. The plate is incubated at RT overnight. Mouse pups (4–7 days old) are decapitated, and the brains are removed and rinsed 4× in DPBS. The tissue is homogenized using a razor blade and put on ice. Dissociation buffer (200 U papain [Worthington Biochemical LS003126] dissolved in EBSS [Invitrogen], 1× DNase I [Worthington Biochemical LS002007; 500× stock solution prepared by dissolving 12,500 U of DNase I in 1 mL of EBSS]) was added to the brain homogenate, and the dish was placed in a 37°C with 5% CO_2_ for 30 min. Neutralization buffer (20% FBS in DMEM-F-12) is added to the homogenate, moved to a 50 mL conical, and centrifuged at 500 × *g* for 15 min at 4°C. The pellet is resuspended in 10 mL of microglia media (ScienCell Microglia Medium #1901) and strained using a 70 μm cell strainer. The strained cell mixture is centrifuged at 500 × g for 10 min at 4°C. The pellet is resuspended in panning buffer and added to the panning plate (which has been washed 3× with DPBS). The panning plate is incubated at RT for 20 min. The panning plate is rinsed 10× with DPBS. 0.25% trypsin (Corning) was added to the panning plate and incubated for 10 min at 37°C with 5% CO_2_. The panning plate was washed 2× with DPBS, ice-cold microglia medium was added, and the plate was incubated for 2 min on ice. A serological pipet is used to dislodge the cells from the panning plate. Cell suspension is collected and centrifuged at 500 × *g* for 15 min at 4°C. The pellet is resuspended in 500 μL of microglia medium, and the cells are counted. One hundred microliters of a 2 × 10^5^ cell/mL suspension are aliquoted to a glass-bottom 96-well plate. The uptake assay is performed as above after 14 days.

### Capsule induction

Fungal strains were grown overnight in YPD at 30°C, 250 rpm. The cells were washed once each with PBS and DMEM. 1 × 10^6^ cells/mL were added to 24-well tissue culture plates. The plates were incubated for 24 h at 37°C with 5% CO_2_. The cell suspension was collected, washed once with PBS, and resuspended in 50 μL PBS. Six microliters of cell resuspension were added to 4 μL of India Ink, and samples were visualized on a 100× objective of an inverted Zeiss microscope. Images were analyzed with ImageJ software for capsule radius. The equation for capsule radius is: (total cell size including capsule − cell body size)/2. Cell size measurements (DMEM condition) were also taken via ImageJ software. Specifically, the μm distance for each measurement (cell body size and total cell size including capsule) was attained using the “straight” distance tool.

### Cell wall staining

Fungal strains were grown overnight in YPD at 30°C, 250 rpm. The cells were washed twice with PBS. 1 × 10^7^ cells/mL were stained with 50 μg/mL Alexa Fluor 488-conjugated concanavalin A (ConA) and 100 μg/mL CFW in PBS. For eosin Y (EoY), cells were washed one additional time in McIlvaine’s buffer (pH 6.0) and stained with 500 μg/mL EoY in McIlvaine’s buffer. Cells were stained for 30 min at room temperature with gentle shaking. Cells were washed three times with PBS and imaged at 100× using an inverted Zeiss microscope. Fluorescence of ConA, CFW, and EoY was obtained at 488 nm, 405 nm, and 558 nm, respectively. MFI was analyzed using a CellProfiler (97) pipeline. Cell size measurements (YPD condition) were also taken via ImageJ software. Specifically, the μm distance for cell body size was attained using the “straight” distance tool.

### Melanin and urease production

Solid and liquid melanin-inducing media were prepared as described (100). Christensen Urea Agar Base was purchased and prepared as directed (Sigma). For solid medium (melanin and urease), fungal strains were grown overnight in YPD at 30°C, 250 rpm, and harvested in the log phase (OD_600_ 0.6–0.8). Cells were washed twice with PBS, and the OD_600_ of each strain was normalized to 0.25. Five microliters of each normalized strain were spotted on the solid medium, and plates were incubated at 30°C for 72 h.

For liquid medium (melanin), fungal strains were grown overnight in YPD at 30°C, 250 rpm, and harvested in the log phase (OD_600_ 0.6–0.8). Cells were washed twice with PBS, and the OD_600_ of each strain was normalized to 0.25 in a 5 mL culture. Cultures were incubated at 30°C, 250 rpm for 18 h. The cultures were centrifuged at 3,000 x g for 5 min. The OD_475_ of the supernatant for each strain was measured.

### Stress plates

Fungal strains were grown overnight in YPD at 30°C, 250 rpm, and harvested in the log phase (OD_600_ 0.6–0.8). Cells were washed twice with PBS and diluted to 1 × 10^7^ cells/mL. Ten-fold serial dilutions were performed, and 5 μL of each dilution was spotted onto YPD, RPMI, and YPD with 2 mM H_2_O_2_. YPD plates were incubated at 30°C, 37°C, and 37°C with 5% CO_2_ for 48 h. RPMI plates were incubated at 37°C with 5% CO_2_ for 48 h. 2 mM H_2_O_2_ plates were incubated at 30°C for 48 h.

## SUPPLEMENTAL MATERIAL

Supplemental Material msphere.00838-25-s0001.docx Figures S1 and S2.

ASM does not own the copyrights to Supplemental Material that may be linked to, or accessed through, an article. The authors have granted ASM a non-exclusive, world-wide license to publish the Supplemental Material files. Please contact the corresponding author directly for reuse.
