# Supplementary material for: Use of a human immortalized microglia cell line to study recognition, phagocytosis, and intracellular survival of Cryptococcus neoformans
Source: mSphere. 2026 Jan 30;11(2):e00838-25. doi: 10.1128/msphere.00838-25 (PMC12931265; doi:10.1128/msphere.00838-25)
Supplement: Supplemental Material — Figures S1 and S2. [file msphere.00838-25-s0001.docx]

Use of a human immortalized microglia cell line to study recognition, phagocytosis, and intracellular survival of *Cryptococcus neoformans*.

Robbi L. Ross, Kassandra Arias-Parbul, Zane M. Douglass, Katrina L. Adams, and Felipe H. Santiago-Tirado

Supplementary Data:

**Supplemental Fig. S1: Impact of opsonization on fungal engulfment, phagosome maturation, and killing by microglia**

**Supplemental Fig. S2. Representative images of phagosome maturation analysis by immunofluorescence.**

**
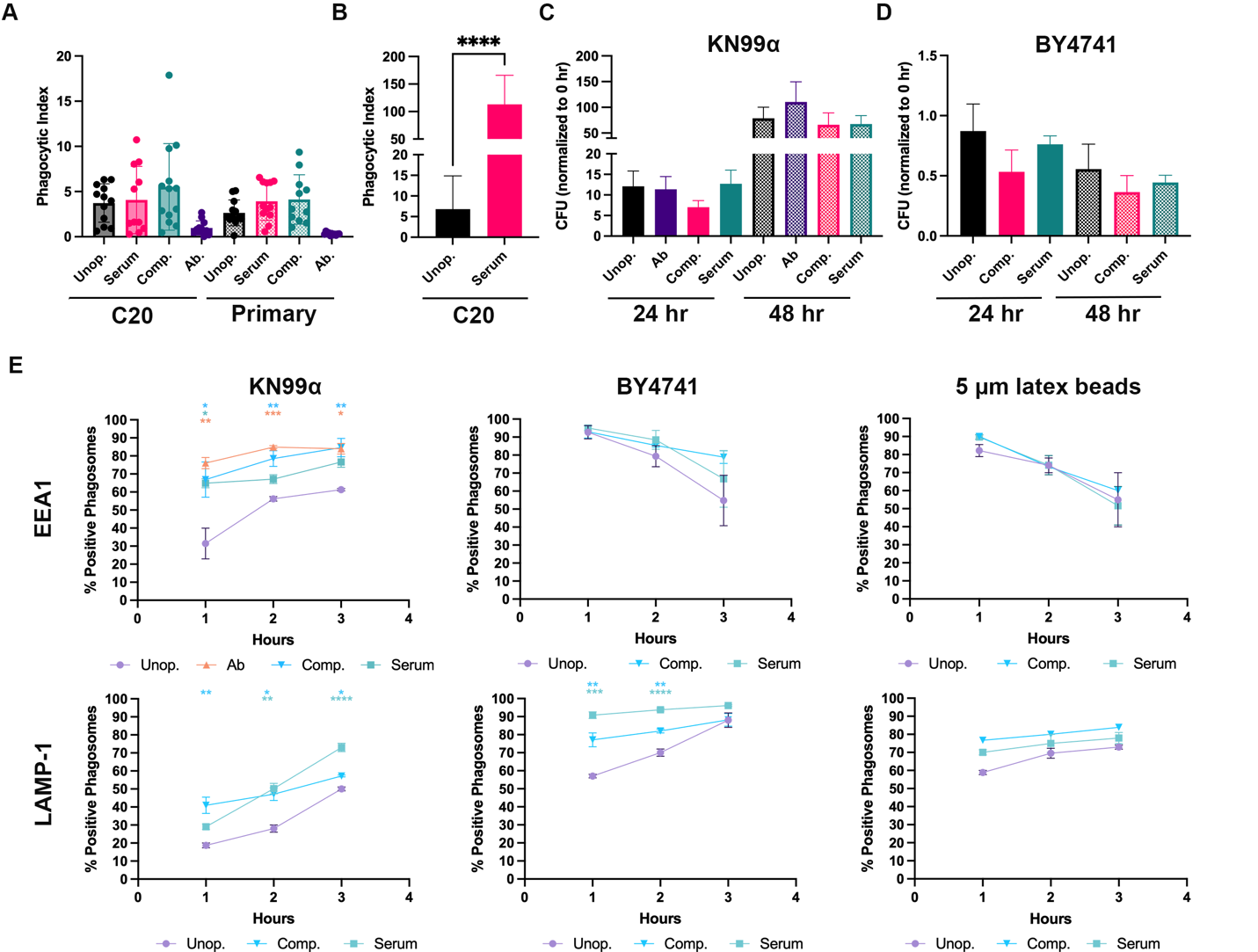
**

**Supplemental Fig. S1. Impact of opsonization on fungal engulfment, phagosome maturation, and killing by microglia.** (A) Two types of human microglia (C20 and primary) were infected with differentially-opsonized KN99α. mCherry-expressing KN99α was unopsonized (Unop) or opsonized with either 40% human serum, 10% human complement (Comp), or 10 μg/mL 18B7 anti-capsule antibody (Ab), and incubated with the different microglia at an MOI of 20:1 for 3 hr. Imaging was performed using automated microscopy and analyzed using a CellProfiler pipeline. Values represent the mean ± SD from three biological replicates. (B) Phagocytosis of unopsonized (Unop.) or serum-opsonized mCherry-expressing BY4741 by C20 cells at an MOI of 20:1 for 3 hr. Imaging was performed using automated microscopy and analyzed using a CellProfiler pipeline. Significance was determined using Welch’s t test, ****, P < 0.0001. Values represent the mean ± SD from three biological replicates. (C and D) *In-vitro* survival of differentially-opsonized KN99α (C) or BY4741 (D) in C20 cells. Fungi and C20 cells were coincubated for 3 hr, at which point one-third of the samples were lysed to determine intracellular fungi, and the other two-thirds were grown for 24 hr and 48 hr, after which they were also lysed. Shown are the colony forming units (CFUs) that were obtained at each timepoint, normalized to the CFUs of initial engulfment (3 hr). Values represent the mean ± SEM from ten biological replicates. Significance was determined using one-way ANOVA with multiple comparisons (Brown-Forsythe and Welch’s corrected), **, P < 0.005; ***, P < 0.0005. (E) Immunofluorescence analysis of cargo-containing phagosomes that are positive for EEA1 or LAMP-1 association at 1, 2, and 3 hours post-coculture with C20 cells. mCherry-expressing fungi or fluorescent latex beads (5 μm) were differentially opsonized and incubated with microglia at an MOI of 20:1 (fungi) or 10:1 (latex beads) for 1, 2, or 3 hours. Coverslips were fixed, stained with primary and secondary antibodies, and imaged via fluorescent microscopy. The quantified cargo is WT *C. neoformans* (KN99α-mCherry), *S. cerevisiae* (BY4741-mCherry), or 5 μm red fluorescent latex beads (CD Bioparticles) as controls. Values represent the mean ± SEM from three biological replicates. Significance was determined using one-way ANOVA with multiple comparisons (Brown-Forsythe and Welch’s corrected) for each timepoint, *, P < 0.05; **, P < 0.005; ***, P < 0.0005; **** P < 0.0001.

**
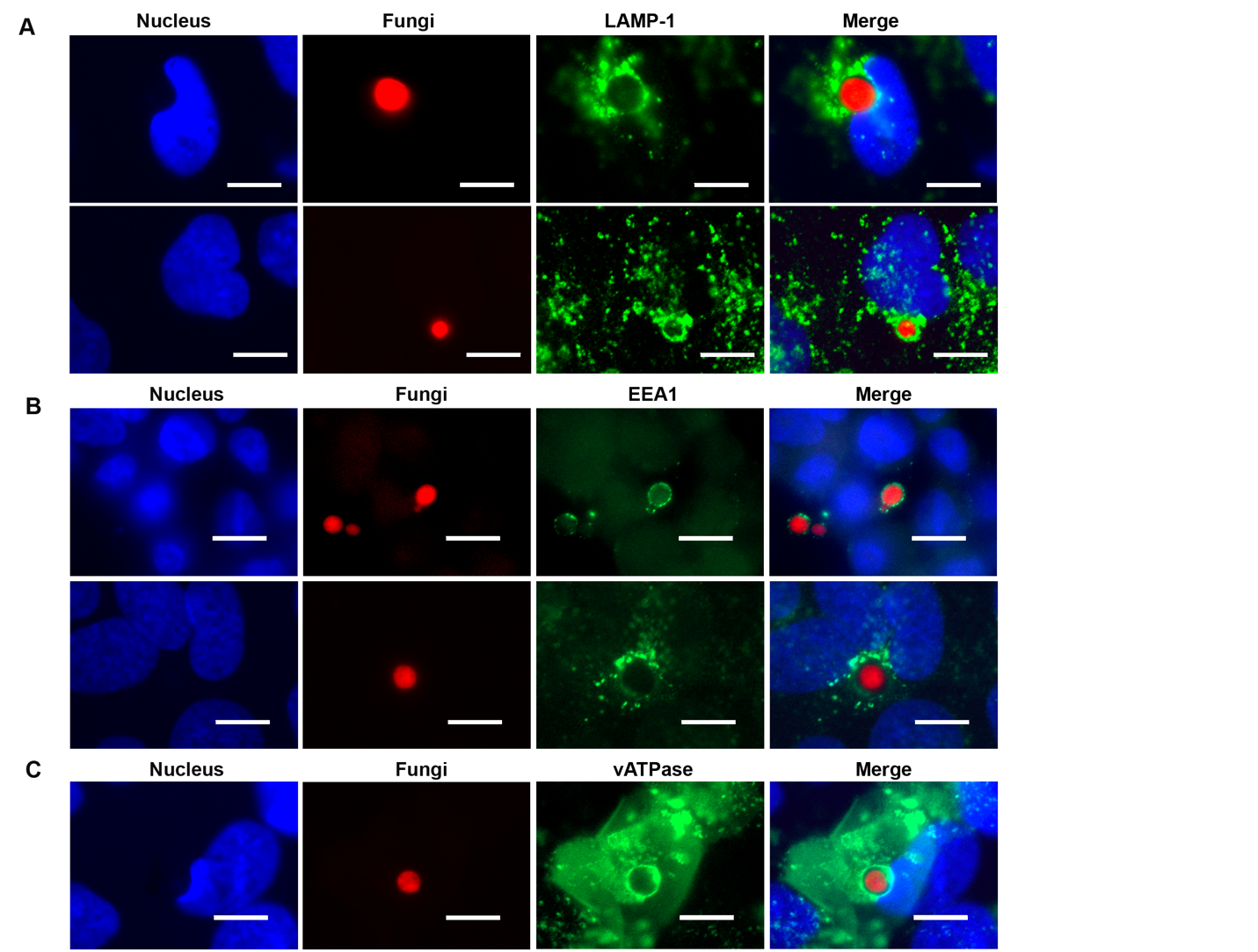
**

**Supplemental Fig. 2. Representative images of phagosome maturation analysis by immunofluorescence.** Representative images showing cryptococcal-containing phagosomes positive for LAMP-1 (A), EEA1 (B), and vATPase (C) association. These images were taken at 100X, with blue showing the nuclei of the C20 cells, red showing intracellular KN99α, and green showing the phagosomal marker. The scale bar is 10 μm.
